# The Genus *Broussonetia*: An Updated Review of Phytochemistry, Pharmacology and Applications

**DOI:** 10.3390/molecules27165344

**Published:** 2022-08-22

**Authors:** Yueru Chen, Lu Wang, Xue Liu, Fulin Wang, Ying An, Wei Zhao, Jinli Tian, Degang Kong, Wenru Zhang, Yang Xu, Yahui Ba, Honglei Zhou

**Affiliations:** College of Pharmacy, Shandong University of Traditional Chinese Medicine, Jinan 250355, China

**Keywords:** *Broussonetia*, phytochemistry, pharmacology, applications

## Abstract

The *Broussonetia* genus (Moraceae), recognized for its value in many Chinese traditional herbs, mainly includes *Broussonetia papyrifera* (L.) L’Hér. ex Vent. (*BP*), *Broussonetia kazinoki* Siebold (*BK*), and *Broussonetia luzonica* (Blanco) Bureau (*BL*). Hitherto, researchers have found 338 compounds isolated from *BP*, *BK*, and *BL*, which included flavonoids, polyphenols, phenylpropanoids, alkaloids, terpenoids, steroids, and others. Moreover, its active compounds and extracts have exhibited a variety of pharmacological effects such as antitumor, antioxidant, anti-inflammatory, antidiabetic, anti-obesity, antibacterial, and antiviral properties, and its use against skin wrinkles. In this review, the phytochemistry and pharmacology of *Broussonetia* are updated systematically, after its applications are first summarized. In addition, this review also discusses the limitations of investigations and the potential direction of *Broussonetia*. This review can help to further understand the phytochemistry, pharmacology, and other applications of *Broussonetia*, which paves the way for future research.

## 1. Introduction

**Figure 1:**
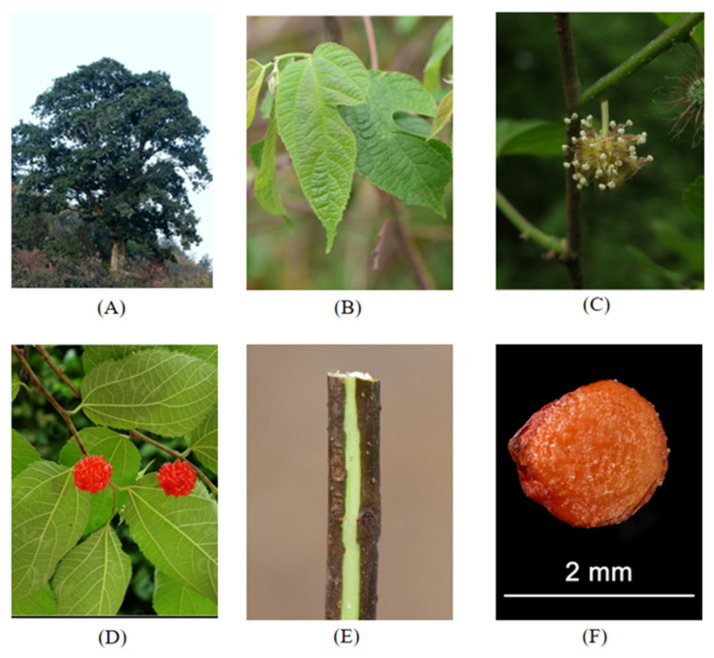
*Broussonetia papyrifera* (L.) L’Hér. ex Vent. Images A–F show, respectively: the whole plant (**A**), leaves (**B**), flowers (**C**), fruits (**D**), twigs (**E**), and seeds (**F**).

**Figure 2:**
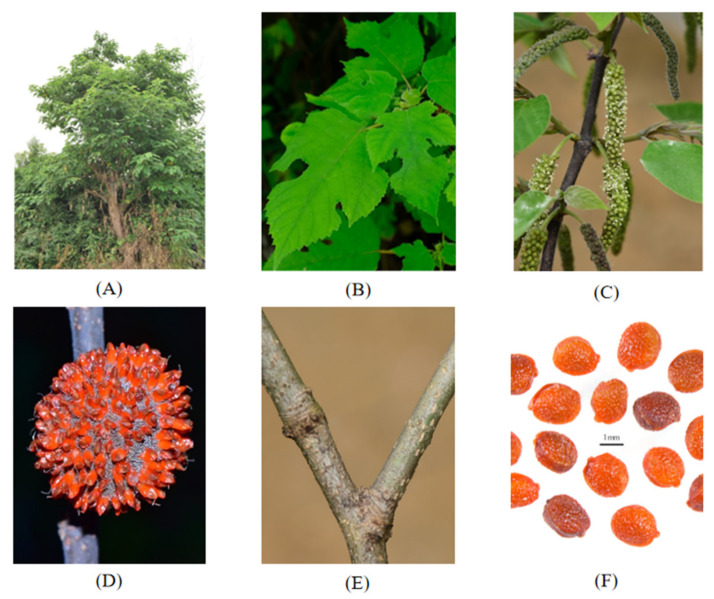
*Broussonetia kazinoki* Siebold. Images A–F show, respectively: the whole plant (**A**), leaves (**B**), flowers (**C**), fruits (**D**), twigs (**E**) and seeds (**F**) of *Broussonetia kazinoki* Siebold.

*Broussonetia* is one of the most significant genera in the Moraceae family, a member of the Urticales order. The genus is composed of eleven species, comprising *Broussonetia papyrifera* (*BP*) (see Figure 1) [1], *Broussonetia kazinoki (BK*) (see Figure 2) [2], *Broussonetia zeylanica* (Thwaites) Corner (*BZ*) [3], *Broussonetia luzonica* (Blanco) Bureau (*BL*) [4], *Broussonetia rupicola* F.T. Wang and Tang (*BR*), *Broussonetia kurzii* (Hook.f.) Corner (*BKU*), *Broussonetia kaempferi* Siebold (*BKA*) [5], *Broussonetia integrifolia* Buch.-Ham. (*BI*), *Broussonetia harmandii* Gagnep*. (BHG*)*, Broussonetia × hanjiana* M.Kim (*BHM*), and *Broussonetia greveana* (Baill.) C.C.Berg (*BG*) [6]. The various *Broussonetia* species have been an excellent source of conventional medicine to treat different diseases. Their roots, barks, fruits, and leaves have all been used in conventional medicine. In China, the leaves have been used to treat chronic prostatitis as a folk medicine [7], as well as for bleeding [8]. The bark could be used for special recipes [8]. The fruits have been confirmed to treat impotence and ophthalmic diseases [7], while the hematochrome from the fruits could be used as a foodstuff in history [9]. In the traditions of Tonga, Fiji, and Samoa, *BP*, a fibrous tree, was the main raw material used to make tapa cloth [10]. Moreover, one of the *Broussonetia* species was used by Cai Lun to create paper, one of the four great inventions of ancient China. *Broussonetia* species were also used as woody forage in ancient history [11].

According to the publication, *Flora of China* [12], the morphology of the genus is described thus: “Trees or shrubs, or vine-like shrubs; there is emulsion, and the winter buds are small. Leaves are alternate, split or non-divided, with serrated margins, basal veins triangular, lateral pinnate veins, and lateral leaves, detached, ovate lanceolate, early fall. The flowers are hermaphroditic or identical; the male flowers are drooping soft inflorescences or spherical cephalic inflorescences, the flowers are indumental, 4 or 3 lobes, the stamens and the flowers are fissured in the same number, folded inward at the time of flower buds, the degenerated stamens are small; the female flowers are densely spherical head-shaped inflorescences, bracts are stick-like, sustenance, flower tube-shaped, apical 3–4 lobes or full margins, succumbs, ovary hidden, stalked, pedunculate lateral, linear, ovules hanging from the ventricular roof. Polyflora is spherical, the embryo is curved, the cotyledons are rounded, flattened, or folded. The genus is distributed in eastern Asia and Pacific islands”.

The diversity of the chemical structures and pharmacological effects has attracted the interest of a variety of researchers. The genus of *Broussonetia* has been found to present 338 compounds, including flavonoids, polyphenols, alkaloids, terpenoids, steroids, and others. Active compounds isolated from *Broussonetia* have been demonstrated to have several biological properties, including antitumor [2], antioxidant [13], anti-inflammation [14], antidiabetic [15], anti-obesity [16], antibacterial [17], and antiviral activities [18], as well as being used for skin whitening [19] and against skin wrinkles, as well as many other uses. In terms of representative applications, *Broussonetia* species could be used as forage for cattle, Hu rams and lambs, growing goats, and other animals because of the high content of protein and fiber [20,21]. In addition, *BP*, combined with *Lonicera japonica,* may be used to treat inflammatory disorders [22]. Liquid bandages that include *Styela clava* tunics and *BK* bark cellulose powders could be used to heal cutaneous wounds [23].

This review efforts to provide comprehensive and up-to-date information on the *Broussonetia* genus, based on published references*,* focusing on phytochemistry, pharmacology, and several other applications. We also discuss the limitations of current research into *Broussonetia*. This review may provide a reference for researchers around the world to investigate and explore the potential applications of the *Broussonetia* genus.

## 2. Phytochemistry

According to the published references, outstanding results have been acquired by a variety of researchers when studying the stems, leaves, barks, radices, fruits, and whole plants of the *Broussonetia* genus. In total, 338 compounds have been isolated from *BP*, *BK*, and *BL* of the *Broussonetia* genus; these compounds consist of 144 flavonoids, 50 penylpropanoids, 38 polyphenols, 35 alkaloids, 17 terpenoids, 5 steroids, and 49 other metabolites. Their chemical structures have been elucidated using the nuclear magnetic resonance spectrometer (NMR) and mass spectrum (MS), along with comparisons with the published data. Their presence might be responsible for the biological properties of the various *Broussonetia* species. However, bioactive compounds that have been confirmed are scarce, the emphasis being on their crude extracts, leading to limitations in the finding of potential candidates for the treatment of corresponding diseases. Given the fact that abundant phytochemicals have been identified, their structure–activity relationships should be unraveled via numerous assays in the future, which will be conducive to unlocking the answers on how phytochemicals develop as targeted bioactive molecules.

### 2.1. Flavonoids

Compounds where the basic core is 2-phenylchromone are classified as flavonoids. Flavones are the main compounds in the 144 flavonoids extracted from *Broussonetia* species. Flavonoids, as primary bioactive compounds, demonstrated the most pharmacological properties, such as antitumor, antioxidant, anti-inflammatory, anti-diabetic, anti-obesity, antibacterial, and antiviral effects, along with skin whitening and anti-wrinkle properties, as well as other activities. In 1994, a new isoprenylated aurone, compound **76,** and a novel isoprenylated flavan, compound **18**, together with the known compounds **38**, **40**, **60,** and **61,** were isolated and characterized from the cortex of *BP* [24]. In 1995, Fang et al. [25] described how two new isoprenylated flavonols, compounds **49** and **51**, were isolated and characterized from the root bark of *BP.* In 1996, Lin et al. [26] reported the presence of compounds **52**, **142**, **143**, and **144** using two-dimensional techniques. A new prenylflavan, compound **37**, and the known compounds **119** and **120** were isolated from the root barks of *BK*, and these compounds were evaluated for cytotoxic activity against several different cell lines [27]. In 2001, an in vitro aromatase inhibition assay was conducted by Lee et al. [28], and the results showed that the novel compounds **59**, **26**, **75**, and **46**, as well as the known compounds **47**, **48**, **50**, **139**, and **140**, were found to be active as aromatase inhibitors, while compounds **56**, **58,** and **63** were inactive. Zhang et al. [29] described, for the first time, five new diprenylated flavonols, compounds **45**, **100**, **136**, **137**, and **138**, which were obtained from an ethanolic extract of the leaves of *BK*; they evaluated the cytotoxic activities of these compounds. In 2002, two new compounds, **71** and **135**, were isolated from the roots of *BP* and the structures were determined using spectroscopy; moreover, compound **71** exhibited significantly inhibitory activities against the PTP1B enzyme [30]. In 2008, the structure of a novel compound, **126**, together with compounds **15**, **16**, **20**, **27**, **69**, **70**, and **110,** were identified by the interpretation of MS, 1H NMR, 13C NMR, HMQC, and HMBC data, and compounds **15**, **20**, and **126** showed high inhibitory activities on mushroom tyrosinase [31]. Ko et al. [32] reported the presence of compounds **4**, **5**, **133**, and **134**, which were isolated from the leaves of *BP*. In 2012, three flavans, compounds **129**, **130,** and **131**, were isolated from the stem barks of *BK* and, in addition, compound **129** significantly inhibited adipocyte differentiation in 3T3-L1 cells [33]. Guo et al. [34] described the new compounds **82** and **83**, together with known compound **128**, which were isolated and purified from an ethyl acetate-soluble extract from the bark of *BP*; moreover, compounds **83** and **128** were found to strongly down-regulate the expression concentrations of estrogen receptor-*α* and inhibit the growth of the human breast cancer line. In the same year, compound **114** was reported for the first time by Ran et al. [35]; this compound showed definite activities against HepG-2. In 2014, the structures of compounds **11**, **12**, **22**, **74**, **78**, and **127** were evaluated, on the basis of NMR spectra analysis and chemical evidence, by Yang et al. [36], and compounds **12** and **127** showed strong antioxidant activity against ABTS and DPPH. In 2018, four new flavans, compounds **116**, **117**, **118**, and **121**, as well as the known compounds **109**, **122**, and **123** were obtained from twigs of *BK* by chiral HPLC resolution; compounds **116**, **117,** and **118** showed the in vitro inhibition of PTP1B [37]. In 2019, compounds **108**, **111,** and **112** were reported by Li et al. [38]. A new isoprenylated flavonol, compound **103**, and the known compounds **104**, **105**, and **106**, were obtained from *Broussonetia* for the first time, being isolated from the twigs of *BP*; compounds **103**–**107** showed significant inhibitory effects on PTP1B [39]. In 2019, the metabolite investigation of root bark extracts of *BP* was reported by Ryu et al. [40]. The results showed that the novel compounds **98** and **99** exhibited anti-inflammatory activity by inhibiting NO production in LPS-induced RAW264.7 cells. Four new compounds (**89**–**92**) and four known compounds (**93**–**96**) were isolated from the root bark of *BP* [41]. Compound **91** showed inhibitory activity against tyrosinase, while compound **92** exhibited cytotoxic activity against three cancer cell lines (NCIH1975, HepG2, and MCF-7), and compound **89** inhibited the production of IL-2 in Jurkat cells [41]. In 2020, the structures of several compounds (**43**, **84**–**88**) were elucidated, based on NMR and HRMS data, showing that compounds **43** and **86** could be used for inflammatory diseases [42]. Compounds **53**, **54**, **55**, **57**, **62**, **64**, **65**, **66**, **67**, **68**, **72**, **77**, **79**, **80** and **81** were reported by Qureshi et al. [43]. In 2021, six previously undescribed prenylated flavonoids, compounds **32**, **33**, **34**, **36**, **39**, and **42**, and three known compounds, **35**, **41**, and **44,** were isolated from the roots of *BK*; compounds **32**, **33**, and **36** showed strong dose-dependent antiosteoclastogenic activities [44]. Compounds **28**–**31** were isolated from the roots of *BK*; compounds **29** and **31** showed anti-inflammatory by inhibiting LPS–induced NO production [14]. In addition, Yadav et al. [45] reported compounds **1**, **3**, **6**, **7**, **8**, **13**, **17**, **19**, **21**, **23**, **24**, and **25**.

All flavonoids are summarized in Table 1, and the structures were summarized in Figure S1.

### 2.2. Penylpropanoids

Compounds with one or several C6-C3 units are classified as penylpropanoids. Several penylpropanoids, such as compound 145 and compound 150, showed anti-tumor and antioxidant activities, respectively.

A total of 50 penylpropanoids have been isolated from *Broussonetia.* In 2009, nine new lignans, compounds **154**, **155**, **172**–**177**, and **184**, and three known lignans, compounds **179**, **182**, and **183,** were isolated from the fruits of *BP*; these compounds exhibited antioxidant activities against H_2_O_2_-induced impairment in PC12 cells [53]. In 2010, Zhou et al. [54] reported that compounds **151**, **170**, **171**, **180**, **181**, and **194** were isolated from *BP* for the first time; these compounds showed antioxidant activity against H_2_O_2_-induced injury in SY5Y cells. In 2014, four compounds (**148**, **165**, **166**, and **178**) were isolated from an n-butanol extract of *BP*, and the structures of these compounds were elucidated on the basis of NMR spectra analysis and chemical evidence [36]. In 2019, Li et al. [38] reported compounds **185**–**192**. In 2020, compounds **162** and **167** were isolated from the CHCl3-soluble part of an ethanolic extract of branches and twigs of *BP* by Malanik et al. [42]. In 2021, Vu et al. [14,44] isolated compounds **156**–**161** from the roots of *BK*. In addition, compounds **145**, **146**, **147**, **152**, and **153** were reported by Yadav et al. [45].

All penylpropanoids are summarized in Table 2, and the structures were summarized in Figure S2.

### 2.3. Polyphenols

Compounds with two or more hydroxyl groups that are not flavonoids, phenylpropanoids, terpenes, or alkaloids are classified as polyphenols. The pharmacological effects of polyphenols, apart from skin whitening and anti-wrinkle properties, have been evaluated by researchers, but this is far from the level of research into flavonoids. 

In total, 38 polyphenols were isolated from *Broussonetia*. In 1999, compounds **203** and **226** were isolated from the root bark of *BK*, and the cytotoxic activity of these compounds was evaluated against several different cell lines [27]. In 2001, Lee et al. [28] reported that compounds **195**, **198**, **199**, **205**, **206**, **223**, and **232** were isolated from the ethyl acetate-soluble extract of the whole plants of *BP*, and compound **199** exhibited to be active as aromatase inhibitors. In 2009, compounds **204**, **207**, **224**, **225**, and **231** were isolated from the methanol extract of *BK*, and the monophenolase inhibition of compounds **204**, **224,** and **225** was determined [55]. In 2018, new polyphenols, compounds **221** and **222**, and known compounds **196** and **210** were isolated from the twigs of *BK*; compounds **196** and **222** showed the in vitro inhibition of protein tyrosine phosphatase 1B [37]. In 2019, compounds **219** and **220** were reported by Li et al. [38]. In addition, the structures of compounds **216**–**218** were elucidated on the basis of spectroscopic data (1D and 2D NMR, MS, MS/MS, and HRMS), and compound **218** exhibited significant inhibitory effects on the NO, iNOS, and pro-inflammatory cytokine production [40]. In 2020, compounds **208**, **209**, **211**, **212**, **123**, **214**, and **215** were reported by Qureshi et al. [43]. In 2021, compounds **197**, **200**, **201**, and **202** were reported by Yadav et al. [45].

All polyphenols are summarized in Table 3, and the structures were summarized in Figure S3.

### 2.4. Alkaloids

Nitrogen-containing organic compounds are classified as alkaloids. Until now, there has been little research on the pharmacological effects of alkaloids.

To date, 35 alkaloids have been isolated from *Broussonetia*. In 1997, Shibano et al. isolated eight new pyrrolidine alkaloids, compounds **253**–**260**, from the branches of *BK*; these compounds demonstrated inhibitory activity on *β*–galactosidase and *β*–mannosidase [58,59]. In 1998, the author also isolated four new pyrrolidine piperidine alkaloids, compounds **261**–**264,** from the branches of *BK* [60,61]. In 1999, three new pyrrolizidine alkaloids, compounds **265**–**267**, were also isolated from the branches of *BK*; these compounds showed the inhibitory activity of glycosidase [62,63]. In 2000, four new pyrrolidine alkaloids, compounds **249**–**252**, showing the ability to inhibit glycosidase were isolated from the branches of *BK* [64]. In 2001, Tsukamoto et al. [65] isolated four new pyrrolidine alkaloids, compounds **244**, **245**, **246**, and **248**, and a new pyrroline alkaloid, compound **247**, from the branches of *BK* [65]. In 2014, two isoquinonline alkaloids, compounds **241** and **242**, were isolated and characterized from the fruits of *BP*; they showed cytotoxic activities on the BEL-7402 and Hela cell lines [66]. In 2020, compounds **238**–**241** were reported by Qureshi et al. [43]. In 2021, compounds **233**–**237** were reported by Yadav et al. [45].

All alkaloids are summarized in Table 4, and the structures were summarized in Figure S4.

### 2.5. Terpenoids and Steroids

Olefins where the molecular formula is an integer multiple of isoprene are classified as terpenoids. Most of the terpenoids are triterpenes. 

Until now, a total of 17 triterpenes have been isolated from *Broussonetia*. Fang et al. reported that two known terpenoids, compounds **283** and **284**, were isolated and characterized from *BP* in 1994 [24] and 1995 [25] respectively. In 2008, three new ent-kaurane type diterpenes, compounds **279**–**281**, were isolated from leaves of *BP*; these compounds showed mild inhibition of tyrosinase and significant inhibition of xanthine oxidase [32]. In 2011, four new euphane triterpenes, compounds **274**–**277**, were isolated from the bark of *BP,* and the structures of these compounds were determined by spectroscopic evidence and chemical methods [69]. In addition, compound **270** was a new tirucallane triterpenoid, and compounds **271**–**273** were isolated from *BP* for the first time. In 2019, compounds **268** and **269** were reported by Li et al. [38]. 

All the terpenoids are summarized in Table 5, and the structures were summarized in Figure S5.

All steroids were isolated only from *BP*. Three compounds (**287**–**289**) were reported by Qureshi et al. [43] in 2020, while compounds **285**–**286** were reported by Yadav et al. [45]. All steroids are summarized in Table 6, and the structures were summarized in Figure S6.

### 2.6. Other Compounds

Apart from the compounds mentioned above, a total of 49 other compounds isolated from *Broussonetia* species are classified as “others”.

Fang et al. [24,25] showed that compounds **329**–**332** were isolated and characterized from the root bark of *BP*. In 2007, two new megastigmane O-glucopyranosides, compounds **327** and **328**, were isolated from the leaves of *BP*; the structures of these compounds were established by chemical methods and spectroscopic techniques, including 2D NMR [72]. In 2010, Zhou et al. [54] established that compounds **323**–**326**, isolated from the fruits of *BP* for the first time, showed antioxidant activity against H_2_O_2_-induced injury in SY5Y cells. In 2011, a novel compound, **322**, and a known compound, **321** were isolated from the n-BuOH extract of *BP* seeds, while their cAMP-regulating activity was evaluated by Mei et al. [73]. In 2014, compounds **316**–**320** were isolated from the n-butanol extract of *BP*, and compounds **317**, **318**, and **320** were found to potently inhibit estrogen biosynthesis in KGN cells [36]. Moreover, compounds **309**–**314** were reported by Yu et al. [51]. In 2016, compounds **333**–**338** were isolated from the ethyl acetate leaf extract of *BL* [71]. In 2019, compounds **293**–**308** were reported by Li et al. [38]. In 2021, compounds 290 and 291 were reported by Yadav et al. [45]. 

All these compounds were summarized in Table 7, and the structures were summarized in Figure S7.

## 3. Pharmacology

Various uses of *Broussonetia* species have inspired researchers’ interest in exploring pharmacological activities by scientific pharmacological assays including in vitro and in vivo. A variety of crude extracts and purified compounds from *Broussonetia* species have been evaluated for different biological effects, such as their antitumor, antioxidant, anti-inflammation, antidiabetic, anti-obesity, antibacterial, and antiviral properties, as well as skin whitening, anti-wrinkle, and other activities. Despite the extensive bioactivities that have been identified, the targeted clinical trials that are normally used to evaluate safety and effectiveness for humans are currently absent. Perhaps the addition of clinical trials might be a more comprehensive and scientific way to ascertain the medical role of the *Broussonetia* genus. All these pharmacological activities are summarized in Table 8.

### 3.1. Anti-Tumor

In 2010, the dichloromethane fraction extracted from the stem barks of *BP* was found to induce apoptosis-related DNA fragmentation; it increased sub-G1 accumulation, increased p53, caspase3, and Bax expression, and inhibited the proliferation of human colon cancer HT-29 cells [74]. The ethanol extract of *BP* exhibited the inhibition of the growth of human osteosarcoma MG63 cells, affected morphological apoptosis, and induced cell-cycle arrest, as found in 2013 [75]. In 2014, seven alkaloids isolated from the ethyl acetate fraction of *BP* fruits at dosages of 1, 5, 10, and 50 mg/mL showed high cytotoxic activities on the BEL-7402 and Hela cell lines, with IC_50_ values of 6.61–47.41 mg/mL and 5.97–40.17 mg/mL, respectively [66]. Zhu et al. [76] explored the mechanism of gastric carcinoma cell SGC-7901 apoptosis, as induced by CALCBP, and the results showed that apoptosis might be related to oxidative stress in the cell mitochondria via the p38-MAPK and ERK-MAPK signal pathways. In an in vitro assay, polyphenols showed significant apoptotic activities on HepG2 cells in a dose-dependent and time-dependent manner by inducing cell cycle arrest at the G1 phase, unregulating the ratio of Bax/Bcl-2 and inhibiting the expression of PKB/AKT and ERK [77].

In 1999, the pure compounds kazinol Q, kazinol R, kazinol D, kazinol K, and 7,4′-dihydroxyflavan, isolated from *BP* root barks, showed strong inhibitory effects on T24, CaSki, PLC/PRF/5, HT3, and SiHa, respectively [27]. In 2011, Wei et al. explored the ability of kazinol Q to induce DNA breakage in the presence of Cu; the results showed that the cell viability of gastric carcinoma SCM-1 cells was significantly decreased [78]. In 2013, (+)-pinoresinol-4′-*O*-*β*-D-glucopyranosyl-4″-*O*-*β*-D-apiofuranoside, apigenin-6-C-*β*-D-glucopyranside, and liriodendrin, isolated and purified from *BP* leaves, exhibited inhibitory effects on HepG-2 cells during the dosage of 100 mmol·L−1; their IC_50_ values were 17.19, 14.56, and 19.53 μg/mL, respectively [35]. Moreover, broussoflavonol B restricted the growth of breast cancer SK-BR-3 cells and breast cancer MDA-MB-231 cells at sub-micromolar concentrations via inducing cell-cycle arrest at the G0/G1 and G2/M phases and inducing the differentiation of cells [79]. In the same year, broussoflavonol B and 5,7,3′,4′-tetrahydroxy-3-methoxy-8,5′-diprenylflavone were prepared from an ethyl acetate-soluble fraction of *BP* barks, exerting potent antiproliferation activities on the ER-positive MCF-7 cells, with IC_50_ values of 4.41 and 4.19 mM, respectively [34]. The two compounds could also inhibit tumor proliferation on BCAP-37 cells in vivo, from a dosage of 0–25 µM [34]. In 2016, kazinol A showed cytotoxicity in T24 and T24R2 cells from a dosage of 0–50 µM via G0/1 arrest, mediated by decreasing cyclin D1 and increasing p21 [80]. In addition, kazinol E was a targeted molecule for breast cancer stem-like cells from a dosage of 0–50 µM, by blocking EGF-induced ERK activity directly [81,82]. Moreover, in 2017, Kim et al. identified that marmesin eliminated mitogen-stimulated proliferation and invasion in both p53 wild-type A549 and p53-deficient H1299 NSCLC cells [83]. In 2018, Park et al. reported that broussochalcone A showed high cytotoxic activities in human hepatoma HepG2 and SK-Hep1 cells, with an IC_50_ value of 20 µM from a dosage of 0–40 µM; these activities were due to cell-cycle arrest by increasing FOXO3, regulating the cell cycle, and activating pro-apoptotic proteins [84]. In another study conducted by Shin et al. in 2019, broussochalcone A also exerted strong cytotoxic effects upon colon and liver cancer cells with a dosage of 0–20 μM, by promoting the phosphorylation/ubiquitin-dependent degradation of *β*-catenin [85]. In 2019, broussoflavonol K showed stronger inhibitory effects on NCI-H1975, MCF-7, and HepG2 than isolicofavonol, with IC_50_ values ranging from 0.90 to 2.00 μM, which were due to cyclization between the isoprenyl moiety and the adjacent phenolic hydroxyl group [41]. A recent study in 2020 investigated the anti-tumor effect of broussoflavonol B; the results showed that it significantly repressed the proliferation of human pancreatic cancer PANC-1 cells, by inactivating the ERK/c-Myc/FoxM1 signaling pathway, with a dosage of 0–100 μM [86]. In 2021, Vu et al. studied the inhibitory effects of eriodictyol, apigenin, and kaempferol against HL-60 cells, with IC_50_ values ranging from 46.43 to 94.06 μM [14]. In a study in 2022, marmesin also exerted cytotoxicity on esophagus cancer cells via inhibiting the PI3K/Akt pathway [87]. From this evidence, it is clear that AMPK is a major regulator of energy metabolic pathways and plays an important role in the regulation of autophagy. In this work, the active compound kazinol C, isolated from *BK* whole herbs or root barks, could markedly induce apoptosis in colon cancer cells by activating AMPK phosphorylation [2,88].

### 3.2. Anti-Oxidant Activity

Excessive oxidative stress is harmful to cells, protein, DNA, and others, so antioxidants are important molecules that can protect humans from this danger. Various assays of antioxidant activity have been used to test these properties, such as DPPH, ABTS, CAA, hydrogen peroxide scavenging activity assays, hydroxyl radical scavenging activity assays, FRAP, lipid peroxidation inhibitory activity, mitochondrial swelling assays, chelation of metal ions (Fe^2+^) assays, xanthin oxidase inhibitory activity assays, hydroxyl radical scavenging activity, superoxide anion free radical scavenging activities, superoxide anion radical scavenging activity assays, ferrous ion chelating capacity assays, and TEAC.

The antioxidant activities of the crude extracts of *Broussonetia* species were measured via the methods mentioned above, of which the most frequently used methods were DPPH, ABTS, FRAP, and hydroxyl radical scavenging activity assays. In 2014, DPPH and pyrogallol autoxidation assays showed that the hydroxyl radical inhibition rate of the seed oil of *BP* was 91.21% [89]. In the same year, the ethanol extract of *BP* fruits was revealed to demonstrate antioxidant activity (0–400 mg/mL), with an IC_50_ value of 155.7 µg/mL for lipid peroxidation inhibition on liver homogenate [90]. In 2013, the ethanolic extract of *BP* fruits showed maximum antioxidant activity by DPPH assay during the dose of 0–600 µg/mL, with an IC_50_ value of 156.3 µg/mL [75]. In 2012, Sun et al. indicated that the ethanol extract from *BP* flowers showed more potent radical scavenging activity than the water extract, which showed 62.88% in the DPPH assay at 5 mg/mL and 61.15% in terms of chelation Fe^2+^-activity at 6 mg/mL [91]. In another experiment, Sun et al. reported that the ethanol and water extracts of *BP* fruits showed strong DPPH radical-scavenging activity at 87.17 ± 0.18% and 58.11 ± 0.11%, respectively [7,91]. The Fe^2+^-chelating activity was approximately 77.51% and 48.26% from an aqueous extract of 5 mg/mL and an ethanol extract of 5 mg/mL [7,91].

Several pure compounds of *Broussonetia* species also showed antioxidant effects in vitro, apart from the extracts mentioned above. In 2020, broussoflavonol A, 5,7,3′,4′-tetrahydroxy-3-methoxy-8,5′-diprenylflavone and kazinol M isolated from *BP* branches and twigs have shown good antioxidant activities, with CAA values of 25.9, 6.4, and 5.4, respectively [42]. In 2014, luteolin, luteoloside, orientin, and isoorientin showed strong radical scavenging activities by DPPH from a dosage of 0.1–3 mg/mL, with SC50 values of 19.72, 19.67, 18.86, and 19.33 mmol/L, respectively [36]. In 2012, Ryu et al. indicated that broussochalcone A and 3,4-dihydroxyisolonchocarpin, isolated from *BP* roots, showed the highest antioxidant activities within a dosage of 0.1–1000 µM, with IC_50_ values of 27.6 ± 0.3 µM and 21.8 ± 0.2 µM through DPPH and IC_50_ values of 5.8 ± 0.1 µM and 7.7 ± 0.4 µM through ABTS, as well as IC_50_ values of 0.6 ± 0.04 µM and 1.8 ± 0.1 µM through an XOD assay [57]. In 2010, curculigoside C, ferulic acid, dihydroconiferyl alcohol, and 3,4-dihydroxybenzoic acid were revealed to have antioxidant activities within a dose of 0.16 to 100 mM, with the IC_50_ values of 39.5, 58.9, 65.3, and 65.6 mM, respectively [54]. In 2009, MTT and DPPH assays showed that erythro-1-(4-hydroxy-3-methoxyphenyl)-2-{4-[(*E*)3-hydroxy-1-propenyl]-2-methoxyphenoxy}-1,3-propanediol, isolated from *BP* fruits (0.16–100 µM), possessed significant antioxidant activities with an IC_50_ value of 60.9 µM [53]. In a diphenyl-2-picrylhydrazyl assay system, broussochalcone A exerted stronger radical scavenging activity within a dose of 1–30 µM than *α*-tocopherol at IC0.200 values of 7.6 ± 0.8 µM [92]. Broussoflavonol F, broussoflavan A, broussoaurone A, and broussoflavonol G inhibited the Fe^2+^-induced formation of TBARS in a concentration-dependent manner, with IC_50_ values of 2.1, 2.7, 1.0 and 1.2 µM, respectively [46].

From Table 8, it can be seen that a plethora of investigations revealed that the roots and leaves possessed stronger antioxidant activities than other parts [93]. Moreover, many assays indicated that the antioxidant activities of bark extracts were superior to wood extracts [94].

### 3.3. Anti-Inflammation

In 2001, broussochalcone A, isolated from *BP* at a dose of 1–20 μM could inhibit NO production in LPS-activated macrophages by inhibiting IkBa phosphorylation, IkBa degradation, nuclear factor-kappa B activation, and iNOS expression, with an IC_50_ value of 11.3 mM [92]. In 2003, papyriflavonol A, isolated from *BP*, was demonstrated to inhibit human group IIA and V sPLA2s dose-dependently and reduce IgE-dependent passive cutaneous anaphylaxis in rats from a dose of 0–250 μM with IC_50_ values of 3.9 to 4.5 mM, suggesting that it could be a novel anti-inflammatory drug in the future [95]. In 2019, Huang et al. demonstrated that broussonin E, isolated from the bark of *BK*, could treat inflammatory diseases by modulating the activation state of macrophages by suppressing ERK and p38 MAPK and enhancing the JAK2-STAT3 signaling pathway [96]. In the same year, flavanone, broussochalcone C, broussoflavanonol A, kazinol V, kazinol W and broussoflavonol B, isolated from root bark in 100% methanol, were shown to have potent anti-inflammatory effects on LPS-stimulated RAW264.7 cell through downregulating iNOS, COX-2, and TNF-α expression within the dose of 1.25–40 μM [40]. In the same year, the anti-inflammatory effect of broussoflavonol H was studied by Tian et al.; the results showed that the compound could significantly suppress the production of IL-2 in Jurkat induced by PHA and PMA, with an IC_50_ value of 9.95 μM [41]. Moreover, in 2020, 5,7,3′,4′-tetrahydroxy-3-methoxy-8,5′-diprenylflavone, kazinol M, broussoflavonol B, broussoflavonol A, and broussofluorenone C, isolated from the branches and twigs of *BP*, showed anti-inflammatory effects by activating NF-κB/AP-1 [42]. In 2021, eriodictyol, apigenin, and kaempferol reduced LPS-induced iNOS expression within the dosage of 0–30 μM in a dose-dependent manner, with IC_50_ values of 11.98, 10.16, and 24.06 μM, respectively [14].

In 2008, the ethanol extract of *BP* roots was shown to reduce abdominal Evan’s blue extravasations, including serotonin and sodium nitroprusside, caused by inflammatory mediators; the effects might be related to inhibiting the vascular permeability via autocrines and NO [97]. In 2010, the anti-inflammatory effect of methanol extract of *BP* heartwood was investigated in NC/Nga mice induced by an extract of the house-dust mite, *Dermatophagoides farina*; the results showed that the methanol extract could obviously inhibit AD-like skin lesions by decreasing the levels of IgE and IL-4 and inhibiting the induction of TARC/CCL17, MDC/CCL22, and RANTES/CCL5 in HaCaT cells [98]. Furthermore, it was reported that the *n*-hexane fraction and *n*-butanol fraction of the methanol extract of *BP* stem bark at a dosage of 10–80 μg/mL were found to have significant anti-inflammatory activities in RAW 264.7 cells by inhibiting NO and pro-inflammatory cytokine production [74,97]. In 2014, the ethanol extract of *BK* leaves within a dose of 200–1000 μg/mL could treat Nc/Nga mice that were predisposed to develop AD-like skin lesions induced by *D. farinae* extract; further study demonstrated that its mechanism might be related to significantly downregulating the plasma levels of IgE and IL-4, as well as inhibiting hTARC secretion in HaCaT cells by activated TNF-α/IFN-γ [97].

In general, the anti-inflammatory activity of *Broussonetia* species was mainly studied in a murine macrophage RAW264.7 cell model and in mice stimulated with LPS. Moreover, the mechanism of anti-inflammatory activity was mainly concentrated on inhibiting NO production and iNOS expression. iNOS was primarily found in macrophages induced by LPS or cytokines to produce a high level of NO as a pro-inflammatory mediator [49]; therefore, the inhibition of NO production or iNOS expression was a critical strategy for the treatment of inflammatory diseases.

### 3.4. Anti-Diabetic and Anti-Obesity Effects

Diabetes is a chronic disease that presents as high levels of glucose in the blood, which may be caused by insulin deficiency and insulin resistance. All in vitro and in vivo studies have demonstrated the antidiabetic effects of different extracts and compounds prepared from *Broussonetia* species.

In 2008, Cha et al. indicated that the ingestion of stem bark powder from *BK* decreased the serum levels of glucose, fructosamine, triglyceride, and total cholesterol, as well as the activity of ALT in the genetically diabetic OLETF rats; the important regulatory factor would be the increased blood insulin level in the animal model [16]. In 2010, Ryu et al. showed that broussochalcone A, papyriflavonol A, broussochalcone B, kazinol A, kazinol B, and 8-(1,1-dimethylallyl)-5′-(3-methylbut-2-enyl)-3′,4′,5,7-tetrahydroxyflanvonol have inhibitory effects against α-glucosidase with a dose of 0.01–1000 μM; the IC_50_ values were 5.3, 11.1, 12.0, 26.3, 3.6, and 2.1 μM, respectively [56]. Moreover, kazinol U [99], isokazinol D, and kazinol C [100] showed therapeutic potential in delaying pancreatic β-cell destruction in type 1 diabetes by blocking the NF-kB pathway in pancreatic β-cells and reducing RINm5F cell damage. A report in 2012 indicated the anti-obesity effect of broussonone A, as well as of other isolated phenolic compounds isolated from *BK* stem barks, the mechanism being related to noncompetitive inhibitory activity on pancreatic lipase, with an IC_50_ of 28.4 µM and an inhibitory effect of adipocyte differentiation in 3T3-L1 cells [33]. In 2016, antidiabetic activity was also observed in mouse 3T3-L1 preadipocyte cells and C2C12 myoblast cells. Lee et al. demonstrated that kazinol B, isolated from *BK* roots, within a dose of 0–20 μM could increase insulin sensitivity via improving glucose uptake through the insulin-Akt signaling pathway, along with AMPK activation [101]. In 2020, treatment with broussoflavonol B and kazinol J in HFD-fed C57BL6 male mice within the dosage of 0–100 μg/mL, showed therapeutic potential in obesity and type 2 diabetes via suppressing pro-inflammatory responses by activating AMPK in 3T3-L1 adipocytes [102]. In addition, an ethanolic extract of *BK* fruits could treat β-cell damage by preventing STZ-induced oxidative stress and suppressing β-cell apoptosis via inhibiting Erk phosphorylation, as found in mice injected with STZ [15], and it could also treat diabetic nephropathy via the activation of Nrf2 and provide protection against PA-induced lipotoxicity in the mesangial cells in diabetes [103].

In a word, the mechanism of anti-diabetic effects is mainly related to blocking the NF-kB pathway and inhibiting *α*-glucosidase activity. The generation of NO via iNOS and reactive oxygen species plays an important role in pancreatic *β-*cell damage. The NF-kB transcription factor was activated by oxidative stress due to reactive oxygen species as well as regulating iNOS expression. Thus, the NF-kB pathway can protect the *β*-cell from damage [99].

### 3.5. Antibacterial and Antiviral Effects

Some studies have shown that the extracts or pure compounds of *Broussonetia* species could suppress bacteria. In 2015, N. Naveen Kumar et al. [104] reported that the hexane extract of *BP* seeds showed high inhibitory activity on *Staphylococcus aureus*, *Proteus vulgaris*, *Bacillus cereus*, and *Enterobacter aerogenes*, whereas it had no inhibitory effect on fungal strains [104]. In 2017, Park et al. analyzed the antibacterial activity of papyriflavonol A within the dosage of 1–1000 µM; the results showed that the potent inhibitory effect of PLpro, with an IC_50_ value of 3.7 μM, along with a further study, showed that it may be a potential anti-COVID-19 agent [105]. Geng et al. [17] indicated that 5,7,3′,4′-tetrahydroxy-3-methoxy-8,5′-diprenylflavone, isolated from the *BP* air-dried aerial part, showed more antibacterial activity in suppressing *Actinomyces naeslundii* and *Porphyromonas gingivalis* (MIC = 1.95 ppm) than the positive control, triclosan, at a dosage of 0.12–250 ppm. In 2021, Ghosh et al. [18] found that six polyphenols (broussochalcone A, papyriflavonol A, 3′-(3-methylbut-2-enyl-3′,4′,7-trihydroxyflavane, broussoflavan A, kazinol F, and kazinol J) showed greater Mpro inhibitory effect than two repurposed drugs (lopinavir and darunavir) and may serve as promising anti-COVID-19 drugs.

### 3.6. Skin Whitening and Anti-Wrinkle Activities

In 2019, Lim et al. [19] reported that kazinol U, a constituent of *BK* root barks, could attenuate melanogenesis within a dose of 0–20 μM via inhibiting MITF expression, inactivating target genes such as tyrosinase, Tyrp1, and Tyrp2, and activating AMPK and MAPK proteins in both in vitro and in vivo experiments. In the same year, it was reported that collagen is the major structural protein in the extracellular space of the connective tissue of the skin, and *BK* stem extract could maintain skin collagen content via inactivating the reactive oxygen species and inhibiting collagenase activity [106].

### 3.7. Other Properties

Out of these pharmacological activities displayed above, *Broussonetia* species also showed the treatment of bone diseases, liver protection, promoting hair growth, anti-angiogenic activities, anticholinesterase effects, increasing cAMP, immune-stimulating activity, and antinociceptive.

In 2021, Vu et al. observed the antiosteoclastogenic activity of broussonols F, G, and K; the results showed that the compounds significantly inhibited RANKL-induced osteoclast formation with a dosage of 10–30 μM in RAW264.7 cells and they may be the lead compounds against bone diseases in the future [44]. In 2020, CSZ extract increased liver function and alleviated DILI in rats induced in acetaminophen (APAP) via regulating the TLR3/JNK/c-jun/c-fos/JAK/STAT3 pathway [107]. In 2020, Lee et al. [108] showed that ethanolic extract of *BP* had the capacity of promoting hair growth by regulating β-Catenin and STAT6 target proteins in human hair follicle dermal papilla (hHFDP) cells within a dose of 0–20 µg/mL. In 2014, the ethanolic extract of *BK* twigs was found to have anti-angiogenic activities with a dosage of 0.1–10 µg/mL; the mechanisms were the inhibition of VEGF-A, stimulated by the phosphorylation/activation of ERK, Akt, and p70S6K, and the downregulation of VEGFR-2 and MMP-2 in human umbilical vein endothelial cells [109]. In 2012, three prenylated flavonols, 8-(1,1-dimethylallyl)-5′-(3-methylbut-2-enyl)-3′,4′,5,7-tetrahydroxyflanvonol,papyriflavonol A, and broussoflavonol B, isolated from *BP* roots, suppressed two human cholinesterases related to Alzheimer’s disease (AD) in a dose-dependent manner, with IC_50_ values ranging from 0.8 to 3.1 μM and from 0.5 to 24.7 μM against HAChE and BChE, respectively [47]. In the next year, the immune-stimulating activity of mice that were immunized intraperitoneally with OVA/alum (100 μg/200 μg) was tested; the results showed that mice given *BK* water extract orally for 21 days could enhance the Th1 immune response and showed no cytotoxicity in the model system [110]. In 2010, Lee et al. [50] explored the estrogenic activity of broussonin A, (+)−(2R) kazinol I, tupichinol C and kazinol U, which showed that these compounds could regulate the E2-responsive genes as functional ER ligands such as E2 in the ER-sensitive MCF-7 cells at 10 µM. Kazinol P, a natural compound isolated from *BK*, showed the therapeutic effects of improving muscle regeneration and repair with the mechanism of promoting myogenic differentiation through activating p38MAPK and MyoD transcription activities [111]. In a study in 2003, Kazinol B, an isoprenylated flavan, showed significant inhibitory activities of nitric oxide (NO) in lipopolysaccharide-activated macrophages, with an IC_50_ of 21.6 mM [112].

**Table 8:** Pharmacological effects of *Broussonetia* species.

	Variety	Parts	In Vivo/In Vitro	Model	Active Components	Dosage	Results	References
Anti-tumor	*BK*	-	in vitro	Colon cancer cells	Kazinol C	0–30 μM	Inducing apoptosis by activating AMPK.	[2]
	*BK*	Roots	in vitro	Hela, HL-60, MCF-7 cells	Eriodictyol, apigenin and kaempferol	-	Cytotoxic activity against HL-60 cells (IC_50_ = 46.43–94.06 μM) and apigenin was cytotoxic against Hela cells (IC_50_ = 49.26 μM).	[14]
	*BP*	Barks	in vitro	HepG2 cells	Polyphenols	0–500 μg/mL	Induced mitochondria-mediated apoptosis by inactivating ERK and AKT signaling pathways.	[77]
	*BK*	Stem barks	in vitro	PANC-1 cells	Broussoflavonol B	0–100 μM	Repressing proliferation by inactivating the ERK/c-Myc/FoxM1 signaling pathway.	[86]
	*BP*	Barks	in vitro	MDA-MB-231 cells	Broussoflavonol B	0–1 µM	Inducing the arrest of the cell cycle and cell death.	[79]
	*BP*	Barks	in vitro	SK-BR-3 cells	Broussoflavonol B	0–1 µM	Inhibiting growth and inducing differentiation of stemlike SK-BR-3 cells.	[113]
	*BP*	-	in vitro	Colon and liver cancer cells	Broussochalcone A	0–20 μM	Cytotoxicity by promoting phosphorylation/ubiquitin-dependent degradation of *β*-catenin.	[85]
	*BP*	Root barks	in vitro	NCI-H1975, HepG2 and MCF-7	Broussoflavonol K	-	IC_50_ = 0.90–2.00 μM	[41]
	*BP*	Barks	in vitro	SGC-7901 cells	Chlorogenic acid-like compounds	50, 100 and 200 μg/mL	Inducing apoptosis through p38-MAPK and ERK-MAPK signaling pathways.	[76]
	*BP*	-	in vitro	HepG2 and SK-Hep1 cells	Broussochalcone A	0, 2.5, 5, 10, 20 and 40 µM	Cell cycle arrest by increasing FOXO3 and cell cycle regulatory and pro-apoptotic proteins (IC_50_ = 20 µM).	[84]
	*BK*	-	in vitro	Esophagus cancer cells	Marmesin	-	Inhibiting the PI3K/Akt pathway.	[87]
	*BK*	-	in vitro	NSCLC cell	Marmesin	0–10 µM	Abrogating mitogen-stimulated proliferation and invasion.	[83]
	*BP*	Roots	in vitro	T24 and T24R2 cells	Kazinol A	0–50 µM	Cytotoxicity through G _0/1_ arrest mediated by cyclin D1 decrease and p21 increase.	[80]
	*BK*	Roots	in vitro	MCF-7 cells	Kazinol E	0–50 µM	Blocking EGF-induced ERK activity.	[81]
	*BK*	Root barks	in vitro	MCF-7 cells	Kazinol E	-	Inhibiting Erk activity by binding the ATP-binding pocket of Erk-1.	[82]
	*BK*	Root barks	in vitro	HT-29 colon cells	Kazinol C	0–120 µM	Promoting AMPK phosphorylation and attenuating HT-29 colon cancer cell growth and viability.	[88]
	*BP*	Fruits	in vitro	A375, BEL-7402 and Hela cells	Total alkaloids and seven individual alkaloids	50, 10, 5, and 1 mg/mL	IC_50_ = 6.61–47.41 mg/mL (BEL-7402 cell line) and IC_50_ = 5.97–40.17 mg/mL (Hela cell line).	[66]
	*BP*	Leaves	in vitro	HepG-2 cells	(+)-pinoresinol-4′-*O*-*β*-D-glucopyranosyl-4″-*O*-*β*-D-apiofuranoside, liriodendrin, apigenin-6-C-*β*-Dglucopyranside	100 mmol/L	IC_50_ were 17.19, 14.56 and 19.53 μg/mL respectively.	[35]
	*BP*	Fruits	in vitro	MG63 cells	Ethanol extract	0–7000 µg/mL	Inhibiting the proliferation associated with apoptosis and cell cycle arrest.	[75]
	*BP*	Barks	in vitro	MCF-7 cells	5,7,3′,4′-Tetrahydroxy-3-methoxy-8,50-diprenylflavone and broussoflavonol B	0–25 µM	Showing high anti-proliferation activities with IC_50_ values of 4.41 and 4.19.	[34]
	*BK*	-	in vitro	SCM-1 cells	Kazinol Q	0–120 µM	Enhancing subsequent cell death due to necrosis.	[78]
	*BP*	Stem Barks	in vitro	HT-29 cells	Dichloromethane Fractions	50, 100, 150, or 200 μg/mL	Inducing apoptosis through p53-dependent mitochondrial signaling pathway.	[74]
	*BK*	Roots barks	in vitro: Human hepatoma,	PLC/PRF/5, T24 cells, human cervical carcinoma, HT-3, SiHa and CaSki cells	kazinols Q, and R, kazinol D, K, H, 7,4′-dihydroxyflavan	-	Showing the great inhibitory effect to T24, CaSki, PLC/PRF/5, HT3 and SiHa respectively.	[27]
Anti-oxidant activity	*BP*	Barks	in vitro	-	Ethanol extracts	-	IC_50_ value was 0.33 ± 0.08 mg/mL	[13]
	*BP*	Branches and twigs	in vitro	THP-1 cells	5,7,3′,4′-tetrahydroxy-3-methoxy-8,5′-diprenylflavone, kazinol M,broussoflavonol A	-	CAA values were 25.9, 6.4, 5.4 respectively.	[42]
	*BP*	Whole plants	in vitro	-	Lignin	10–100 mg/L	Lignin with more phenolic hydroxyl groups.	[114]
	*BP*	Fruits	in vitro	-	Three purified fractions	0–2.0 mg/mL	IC_50_ values of three purified fractions were 0.54, 0.86, and 0.57 mg/mL respectively.	[115]
	*BP*	Leaves	in vitro	KGN cells	Luteolin, luteoloside, orientin, isoorientin	0.1–3 mg/mL	SC_50_ values was 19.72, 19.67, 18.86 and 19.33 mmol/L respectively.	[36]
	*BP*	Seeds	in vitro	-	Seed oil	0.2–0.8 *v/v*	The hydroxyl radical inhibition rate was 91.21%	[89]
	*BP*	Fruits	in vitro	-	Ethanol extract	0–400 mg/mL	IC_50_ for lipid peroxidation inhibition on liver homogenate was 155.7 µg/mL	[90]
	*BP*	Fruits	in vitro	MG63 cells	Ethanolic extract	0–600 µg/mL	DPPH assay showed IC_50_ value of 156.3 µg/mL.	[75]
	*BP*	Flowers	in vitro	-	Ethanol and water crude extracts	5 mg/mL 6 mg/mL	The ethanol extract showing 62.88% in the DPPH radical scavenging method and 61.15% in chelation Fe^2+^-activity.	[91]
	*BP*	Fruits	in vitro	-	Ethanol and water crude extracts	DPPH: 0.5–5 mg/mL Fe^2+^-activity: 0.5–5 mg/mL	DPPH radicals with a percentage inhibition of 87.17 ± 0.18% to ethanol extract and 58.11 ± 0.11% to aqueous extract.Fe^2+^-chelating activity of approximately 77.51% of aqueous extract and the ethanol extract showed a chelation capacity of 48.26%.	[7]
	*BP*	Roots	in vitro	-	Broussochalcone A and 3,4-dihydroxyisolonchocarpin	0.1–1000 µM	IC_50_ values of 27.6 ± 0.3 µM and 21.8 ± 0.2 µM through DPPH assay, which IC_50_ values of ABTS were 5.8 ± 0.1 µM and 7.7 ± 0.4 µM as well as IC_50_ values of XOD were 0.6 ± 0.04 µM and 1.8 ± 0.1 µM.	[57]
	*BP*	Fruits	in vitro	RAW264.7 cells	Petroleum extract	DPPH: 0.31 to 5.0 mg/mL superoxide anion radical scavenging activity: 2.5–40 mg/mL hydroxyl radical scavenging activity: 0.625–10 mg/mL	IC_50_ = 8.20 ± 0.003 mg/mL (DPPH). IC_50_= 89.86 ± 3.40 mg/mL (superoxide anion). IC_50_ =19.63 ± 0.36 mg/mL (hydrogen peroxide).	[116]
	*BP*	Fruits	in vitro	SY5Y cells	3,4-dihydroxybenzoic acid, dihydroconiferyl alcohol, ferulic acid and curculigoside C	0.16–100 mM	The IC_50_ values were 39.5, 58.9, 65.3, and 65.6 mM respectively through a DPPH assay.	[54]
	*BP*	Barks and woods	in vitro	-	Ethyl acetate fractionhexane fraction	-	The antioxidant activity of bark extract was superior to that of wood.	[94]
	*BP*	Radixes and leaves	in vitro	SH-SY5Y cells	Methanol extract	0.1–2.5 mg/mL	*BP* radixes and leaves possessed the best scavenging activities for DPPH, ABTS radical, and H_2_O_2_.	[93]
	*BP*	Fruits	in vitro	PC12 cells	Erythro-1-(4-hydroxy-3-methoxyphenyl)-2-{4-[(*E*)3-hydroxy-1-propenyl]-2-methoxyphenoxy}-1,3-propanediol	0.16–100 µM	IC_50_ =60.9 µM (DPPH assay).	[53]
	*BP*	-	in vitro	RAW 264.7 cells	Broussochalcone A	1–30 µM	IC_0_._200_ was 7.6 ± 0.8 µM (diphenyl-2-picrylhydrazyl assay system).	[92]
	*BP*	Roots	in vitro	-	Broussoflavan A, broussoflavonol F,broussoflavonol G, broussoaurone A	-	Inhibiting the Fe^2+^-induced formation of TBARS with IC_50_ values of 2.1, 2.7, 1.0 and 1.2µM respectively.	[46]
Anti-inflammation	*BK*	Roots	in vitro	RAW264.7 cells	Eriodictyol, apigenin, kaempferol	0–30 μM	Reducing iNOS expression with IC_50_ values of 11.98, 10.16, and 24.06 μM.	[14]
	*BP*	Branches and twigs	in vitro	THP-1 cells	Kazinol M, broussoflavonol B, broussoflavonol A, 5,7,3′,4′-tetrahydroxy-3-methoxy-8,5′-diprenylflavone and broussofluorenone C	1 μM	Activating NF-κB/AP-1.	[42]
	*BP*	Root barks	in vitro	NCIH1975, HepG2, and MCF-7 cells	Broussoflavonol H.	-	Inhibiting the production of IL-2 in Jurkat induced by PHA and PMA (IC_50_ = 9.95 μM).	[41]
	*BP*	Root barks	in vitro	RAW264.7 cells	Flavanone, broussochalcone C, broussoflavanonol A, kazinol V, kazinol W and broussoflavonol B	1.25–40 μM	Reducing NO production through downregulating iNOS, COX-2, and TNF-*α* expression and the expression of iNOS protein.	[40]
	*BK*	Barks	in vitro	RAW264.7 cells	Broussonin E	2.5–20 μM	Inhibiting the ERK and p38 MAPK and enhancing the JAK2-STAT3 signaling pathway.	[96]
	*BK*	Leaves	in vivo	mice	Ethanol extract	200–1000 μg/mL	Down-regulating the plasma levels of IgE and IL-4 and inhibiting hTARC secretion in HaCaT cells by activated TNF-α/IFN-γ.	[117]
	*BP*	Stem barks	in vitro	RAW 264.7 cells	n-hexane fractionof methanol extract	10–80 μg/mL	Inhibiting the NO production and proinflammatory cytokines.	[118]
	*BP*	Stem barks	in vitro	RAW 264.7 cells	n-butanol fraction	0–150 μg/mL	Inhibiting iNOS expression in RAW 264.7 macrophages.	[74]
	*BK*	Heartwood	in vivo	mice	EtOH extract	50–250 mg/mL	Inhibiting IgE production in *β-*cell and mast cell infiltration by IL-4 and chemokines by inhibiting Th2-cell activation by allergens.	[98]
	*BP*	-	in vivo	-	Ethanol extract	-	Inhibiting vascular permeability via autocrines and nitric oxide.	[97]
	*BP*	-	in vitro	Bone marrow cells	Papyriflavonol A	0–250 μM	Inhibiting human group IIA and V sPLA2s with IC_50_ values of 3.9 and 4.5 mM.	[95]
	*BP*	-	in vitro	RAW 264.7 cells	Broussochalcone A	1–20 μM	Inhibiting NO production with an IC_50_ of 11.3 mM via inhibition of IkBa phosphorylation, IkBa degradation, nuclear factor-kappa B activation, and iNOS expression.	[92]
	*BK*	Root barks	in vitro	RAW 264.7 cells	Tupichinol C, kazinol U, kazinol A, kazinol I, broussonin A, kazinol C, kazinol D	0–20µM	Suppressing the LPS-induced high level of NO with IC_50_ values of less than 6 µM and attenuating protein and mRNA levels of inducible iNOS.	[49]
Anti-diabetic and Anti-obesity Effects	*BK*	Fruits	in vivo	mice	Ethanolic extract	-	Inhibiting Erk phosphorylation by preventing STZ-induced oxidative stress and beta cell apoptosis.	[15]
	*BK*	Fruits	in vitro	SV40 MES13 cells	Ethanolic extract	0–40 μg/mL	Ethanolic extract induced the expression of antioxidant enzymes by activating Nrf2 and prevented palmitate-induced lipotoxicity.	[103]
	*BP*	Root barks	in vivo	mice	Broussoflavonol B and kazinol J	0–100 μg/mL	Suppressing pro-inflammatory responses via activating AMPK.	[102]
	*BK*	Root barks	in vivo	mice	Kazinol C and isokazinol D	5–25 μM	Blocking the NF-κB pathway and reducing the extent of *β*-cell damage.	[100]
	*BK*	Stem barks	in vitro	3T3-L1 cells	Broussonone A together with other isolated phenolic compounds	100 µM	Inhibitory activity against pancreatic lipase with IC_50_ of 28.4 µM, and has inhibitory effects on adipocyte differentiation.	[33]
	*BK*	Root barks	in vitro	RINm5F cells	Kazinol U	0–60 μM	Blocking the NF-kB pathway and reducing cells damage.	[99]
	*BP*	Roots	-	-	Broussochalcone A, broussochalcone B, kazinol A, kazinol B, 8-(1,1-Dimethylallyl)-5′-(3-methylbut-2-enyl)-3′,4′,5,7-tetrahydroxyflanvonol and papyriflavonol A	0.01–1000 μM	IC_50_ values of 5.3, 11.1, 12.0, 26.3, 3.6, and 2.1μM respectively.	[56]
	*BK*	Stem barks	in vivo	mice	Stem bark powders	-	Decreasing the serum levels of glucose, fructosamine, triglyceride, and total cholesterol and the activity of ALT, and increasing blood insulin level.	[16]
Antibacterial and Antiviral Effects	*BP*	-	in vitro	-	Broussochalcone A, papyriflavonol A,3′-(3-methylbut-2-enyl)-3′,4′,7-trihydroxyflavane, broussoflavan A, kazinol F and kazinol J	-	These six polyphenols are more potent Mpro inhibitors than two repurposed drugs (lopinavir and darunavir).	[18]
	*BP*	Whole plants	in vitro	-	5,7,3′,4′-tetrahydroxy-3-methoxy-8,5′-diprenylflavone	0.12–250 ppm	Suppressing *Porphyromonas gingivalis* (MIC = 1.95 ppm).	[17]
	*BP*	Roots	in vitro	-	Papyriflavonol A	1–1000 µM	Inhibitory effect of PLpro with an IC_50_ value of 3.7 µM.	[105]
	*BP*	Fruits	in vitro	-	BPP-3	0.4–2.0 mg/mL	The minimum inhibitory concentration of BPP-3 against *E. coli, P. aeruginosa, B. subtilis* and *S. aureus* were 0.3 mg/mL, 0.25 mg/mL, 0.3 mg/mL and 0.25 mg/mL, respectively.	[115]
	*BP*	Seeds	in vitro	-	Hexane extract	0.25%, 0.5%, 1%, 2%, and 4% (*v/v*)	The seed oil has an inhibitory effect on *Staphylococcus aureus, Proteus vulgaris, Bacillus cereus,* and *Enterobacter aerogenes*.	[104]
Skin whitening and Anti- skin wrinkles Activities	*BK*	Root barks	in vivoin vitro	Zebrafish/B16F10 cells	Kazinol U	0–20 μM	Inhibitory activity of MITF and downstream target genes such as tyrosinase, Tyrp1 and Tyrp2.	[19]
	*BK*	Stems	in vitro	HEK-293T cells	EtOH extract	0–100 μg/mL	Maintaining the collagen content of the skin by eliminating reactive oxygen species and inhibiting collagenase activity.	[106]
Others	*BK*	Roots	in vitro	RAW264.7 cells	Broussonol F, G and K	10–30 μM	Inhibiting RANKL-induced osteoclast formation.	[44]
	*-*	Fruits	in vivo	mice	Chushizi	-	Increasing liver function and alleviating DILI via regulating the TLR3/ JNK/ c-jun/c-fos/JAK/STAT3 pathway.	[107]
	*BP*	-	in vitro	hHFDP cells	Ethanolic extract	0–20 µg/mL	Regulating β-Catenin and STAT6 target protein.	[108]
	*BK*	Twigs	in vitro	human umbilical vein endothelial cells	Ethanolic extract	0.1–10 µg/mL	Inhibiting VEGF-A stimulated phosphorylation/activation of ERK, Akt and p70S6K, the downstream targets of the VEGFR-2 signaling pathways, and downregulation of VEGFR-2 and MMP-2.	[109]
	*BP*	Roots	in vitro	-	8-(1,1-Dimethylallyl)-5′-(3-methylbut-2-enyl)-3′,4′,5,7-tetrahydroxyflanvonol, papyriflavonol A and broussoflavonol B	0–30 µM	Inhibiting hAChE and BChE with IC_50_^′^s ranging from 0.8 to 3.1 μM and from 0.5 to 24.7 μM, respectively.	[47]
	*BP*	Seeds	in vitro	N1E-115 cells	Chushizilactam A and adenosine	50 µM	Adenosine could obviously increase cAMP.	[73]
	*BK*	Stems	in vivo	mice	Water extract	-	Water extract has immune-stimulating activity by enhancing the Th1 immune response.	[110]
	*BK*	-	in vitro	MCF-7 cells	Broussonin A, tupichinol C kazinol U and (+)-(2*R*) kazinol I	10 µM	Modulating the E2-responsive genes as functional ER ligands such as E2.	[50]
	*BK*	Roots	in vitro	C2C12 and 10T1/2 cells	Kazinol P	1000 nM	Promoting myogenic differentiation through the activation of p38MAPK and MyoD transcription activities.	[111]
	*BK*	Root barks	in vivo	RAW 264.7 cells	Kazinol B	6.25–50 µM	Inhibiting the NO synthesis with an IC_50_ of 21.6 mM	[112]

## 4. Application

### 4.1. Supplements to the Diet of Animals

It was reported that a supplement of *Broussonetia* species in the roughage diet was beneficial to the growth performance, carcass traits, meat quality and color, immune response, digestibility of crude protein, and rumen fermentation in different animals, such as Hu rams and lambs and growing goats [20,119]. Tao et al. [120] demonstrated that beef cattle fed on a diet with 15% *BP* could enhance their antioxidant functions by decreasing blood 8-OHdG and MDA and increasing blood SOD and TAC; the supplements could strengthen the performance by increasing final BW, ADG, DMI and FCR, improve the meat quality by lowing pH and drip loss and increasing CIE L, and also increase the PUFA and DHA concentrations in meat. A diet supplemented with *BP* could enhance immune and antioxidant function, as well as increase the polyunsaturated fatty acid concentrations in the milk of Holstein cows [121,122]. A diet supplemented with *BP* leaf extracts at a certain dosage could increase the growth performance and antioxidant capacity of weaned piglets, enhance immune functions and disease resistance, reduce the occurrence of diarrhea, and affect the composition of fecal microflora [123].

### 4.2. Phytoremediation of Heavy Metal-Contaminated Soil

The effective phytoremediation of heavy metal-contaminated soil requires species with high metal tolerance. *Broussonetia* species were excellent choices, owing to their adaptation to drought and to a saline-alkali environment [124]. Zeng et al. [125] showed that *BP* could effectively alleviate the adverse effects of heavy metal-contaminated soil on plant growth by enhancing the antioxidant enzyme activities in leaves and binding heavy metal-contaminated soil with organic acids, carbohydrates, protein, and amino acids in roots. Huang et al. [126] isolated *Bacillus cereus* HM5 and *Bacillus thuringiensis* HM7, and explored their potential to improve the effect of remedying Mn pollution by *BP*; the results showed that the biomass, total root length, surface area, crossings, tips, forks, and root activity of *BP* with the two strains were higher than *BP* without the two strains, so the two strains could promote the accumulation of Mn. Luo et al. [127] indicated that *BP* could be used for the revegetation and phytostabilization of zinc-smelting slag sites because of the high heavy-metal tolerance and low heavy-metal accumulation. Co-planting was also a sustainable approach for the phytoremediation of the heavy metal-contaminated soil. The hyperaccumulator *Pteris vittata* L., co-planted with *BP*, could improve the environmental quality of heavy metal-contaminated soil by promoting the growth and uptake of *P. vittata* L. and improving the comprehensive extraction of metal [128]. Zeng et al. [129] selected *Pteris vittata* L., *Arundo donax* L., *Morus alba* L. and *BP* for tree–herb co-planting; the results indicated that the four-herb co-planting system positively affected the soil microbes and had stronger impacts on the composition of soil microorganisms.

### 4.3. Combination Using

The pharmacological effects of *Broussonetia* genus are limited but, when combined with other plants, it can exert more meaningful pharmacological effects. A liquid bandage was made from *Styela clava* tunics and *BK* barks cellulose powders; it could accelerate wound-healing in the surgical skin wounds of Sprague-Dawley rats by stimulating re-epithelialization and connective tissue formation, without liver or kidney toxicities [23]. A new phytoformula containing *BP* and *Lonicera japonica* was revealed to have therapeutic potential for systemic septic inflammation, as well as chronic bronchitis, and against LPS-induced septic inflammation in mice by reducing the induction of some important proinflammatory cytokines, at a dosage of 200–400 mg/kg [22]. *Phellinus linteus*, cultured with *BK*, could inhibit melanogenesis by activating the phosphatidylinositol 3-kinase/Akt/glycogen synthase kinase-3beta signaling pathway and down-regulating the microphthalmia-associated transcription factor [130].

### 4.4. Inhibition of Target Enzymes

*Broussonetia* species showed inhibitory effects on target enzymes, such as monophenolase and diphenolase of tyrosinase [55], mushroom tyrosinase [31], xanthine oxidase, and protein tyrosine phosphatase 1B (PTP1B) [37]. Four compounds, kazinol S, kazinol C, broussonin C, and kazinol F, isolated from *BK* roots, showed inhibitory effects on the monophenolase of tyrosinase, with IC_50_ values ranging between 0.43 and 17.9 µM [55]. They also showed reversible slow-binding inhibitory effects on the diphenolase of tyrosinase, with IC_50_ values of 22.8, 1.7, 0.57, and 26.9 µM, respectively [55]. Zheng et al. [31] revealed the inhibitory activities of mushroom tyrosinase, using L-tyrosine as a substrate of quercetin, broussoflavonol F, 3,5,7,4′-tetrahydroxy-3′-(2-hydroxy-3-methylbut-3-enyl) flavone, and uralenol, with IC_50_ values of 96.6, 49.5, 57.8, and 82.3 µM, respectively; the results were better than arbutin, a well-known tyrosinase inhibitor [31]. Broussonetones A-C, three new ent-kaurane-type diterpenes, showed marginal inhibitory effect against tyrosinase and xanthine oxidase, as indicated by Ko et al. [32]. 3,3′,4′,5,7-pentahydroxyflavone, 8-(1,1-dimethylallyl)-5′-(3-methylbut-2-enyl)-3′,4′,5,7-tetrahydroxyflanvonol, uralenol and broussochalcone A showed the in vitro inhibition of PTP1B, as reported by Chen et al. [30]. Broukazinol A, 7,4′-dihydroxy-3′-prenylflavan, Broukazinol C, (2*S*)-7,3′-dimethoxy-4′-hydroxyflavan, broussonin B, 1-(4-hydroxy-2-methoxyphenyl)-3-(4-hydroxy-3-prenylphenyl) propane and 1-(2,4-dihydroxy-3-prenylphenyl)-3-(4-hydroxyphenyl) propane, isolated from *BK* twigs, also showed the inhibition of PTP1B, as reported by Xue et al. [37].

### 4.5. Papermaking and Cloth-Making

The barks of the *Broussonetia* species were excellent materials for the production of papers and clothes, which can be attributed to the high fiber content of the phloem. Cai Lun papermaking was one of the four great inventions of ancient China; this great invention used *BP* barks at that time. Notably, the first banknote in the world was made from *BP*. Papers made by *BP* barks were once used for making books and for writing in the Tang dynasty and their use continued during the Ming and Qing dynasties, as evidenced by many Dunhuang and Turfan manuscripts [21]. The bark of *BP* can also be used to make ancient tapa clothes; in this process, the barks were peeled from the cut stems to obtain a long strip, then the manufacturer removed the inner bark and washed and pounded the fibers to flatten them, felted them together with sheets in the sun, and finally printed them with native dyes to produce the finished traditional tapa cloth [10].

### 4.6. Others

In addition to the applications mentioned above, the remaining applications are classified as “others”. A study conducted by Qiu et al. explored their application in cleaning up phosphorus pollution; they showed that *BP* biochar, when combined with phosphate in the forms of exchangeable phosphorus, Al-bound phosphorus, and Fe-bound phosphorus, could be used to treat eutrophic bodies of water [131]. Zhang et al. [132] measured the ability of *BP* to resist drought due to the electrophysiological characteristics of the plants; the results showed that the relative tensity of *BP* and *M*. *alba* were 3.965 and 2.624, respectively. The author also demonstrated that the minimal fluorescence efficiency and maximal photochemical efficiency were 5.496 and 7.640 for *BP* and 6.577 and 5.359 for *M*. *alba*, respectively; therefore, the drought-resistance ability of *BP* was greater than that of *M*. *alba*. Mo et al. [133] studied the ability of *BP* to control air pollution; the results showed that *BP* was efficient in capturing small particles and showed high levels of PM accumulation.

## 5. Conclusions and Further Perspectives

This review mainly summarized the phytochemistry, pharmacology, and applications of the *Broussonetia* genus. In this work, we present a list of 338 compounds that have been isolated from the herbs of *Broussonetia,* including 144 flavonoids, 50 phenylpropanoids, 38 polyphenols, 35 alkaloids, 17 terpenoids, 5 steroids, and 49 other metabolites, which indicated that flavonoids were the main constituent in the genus of *Broussonetia*. A variety of pharmacological activities have been demonstrated in vivo or in vitro assays, including anti-tumor, antioxidant, anti-inflammatory, antidiabetic, anti-obesity, antibacterial, and antiviral properties, as well as skin whitening and anti-wrinkle activities. Nevertheless, some studies of the *Broussonetia* genus are limited at present.

First, phytochemistry clarified 338 compounds isolated from *BP*, *BK*, and *BL*, but only 5 steroids and 17 terpenoids were involved. Undoubtedly, we can make more effort toward the exploration of steroids and terpenoids by the targeted phytochemical methods. Second, the *Broussonetia* genus consists of 11 species, but the investigations of phytochemistry, pharmacology and applications were only studied in *BP*, *BK*, and *BL*. Therefore, it is extremely important for researchers to conduct a comprehensive evaluation of other species to extend the available source domain. Third, pharmacological studies have uncovered anti-tumor, antioxidant, anti-inflammatory, anti-diabetic, anti-obesity, antibacterial, and antiviral properties, as well as skin whitening and anti-wrinkle activities. Notably, the relevant bioactive compounds only include flavonoids, penylpropanoids, and polyphenols; therefore, the pharmacological activities of the remaining compounds need to be further explored by researchers. Furthermore, the pharmacological activities of the concrete compounds of crude extracts need to be confirmed in the future, which would be conducive to finding new candidates for corresponding diseases. Fourth, mechanism analysis indicated that active compounds and extracts mainly showed anti-tumor effects by inducing cell apoptosis and triggering cell cycle arrest; they showed anti-inflammatory activity mainly by inhibiting NO production and iNOS expression and showed anti-diabetic effects mainly via blocking the NF-kB pathway and *α*-glucosidase. Undoubtedly, we can see that mechanism studies of the active compounds and extracts isolated from *Broussonetia* species were mainly concentrated on the typical targets and pathways. Hence, new targets and pathways should be detected in the future. Fifth, in vitro models were considered to be the main conditions; therefore, more in vivo models should be used to investigate properties in the future. Sixth, although the applications indicated that *Broussonetia* species could be used to supplement the diet of beef cattle, growing goats, cows, and piglets, and showed multiple beneficial effects on their growth performance, carcass traits, meat quality, and immune response, the concrete clinical safety, toxicity and pharmacokinetics studies for animals and humans were extremely limited; thus, exploring these aspects is the top priority in future research.

Overall, this updated review on the plants of the *Broussonetia* genus can provide an important and valuable reference for researchers interested in *Broussonetia* and promote scientific development and utilization of the *Broussonetia* genus.

## Figures and Tables

**Table 1:** Flavonoids isolated from *Broussonetia* species.

Number	Compounds	Parts	Source	References
1	Gancaonin P	Whole plants	*BP*	[45]
2	Isolicoflavonol	Whole plants	*BP*	[27,30,42,44]
3	Lespedezaflavanone C	Whole plants	*BP*	[45]
4	Vitexin	Leaves	*BP*	[31,35,44]
5	Apigenin	Leaves	*BP/BK*	[13,31,35,44]
6	Pinocembrin	Whole plants	*BP*	[45]
7	Isobavachalcone	Whole plants	*BP*	[45]
8	4-Hydroxyisolonchocarpin	Roots	*BP*	[37,44,45]
9	Luteolin	Leaves/twigs	*BP*	[30,35,44,46]
10	Cosmosiin	Leaves	*BP*	[35,45]
11	Isoorientin	Leaves	*BP*	[36,45]
12	Orientin	Leaves	*BP*	[36,45]
13	2,4,2′,4′-Tetrahydroxychalcone	Whole plants	*BP*	[43,45]
14	Abyssinone II	Whole plants	*BP*	[28,45]
15	Uralenol	Roots/twigs/barks	*BP*	[29,30,33,37,38,44]
16	Papyriflavonol A	Root barks/twigs	*BP*	[30,33,40,44,47,48]
17	Norartocarpanone	Whole plants	*BP*	[45]
18	Broussoflavan A	Root barks	*BP*	[23,39,44,45,49]
19	Dihydrokaempferol	Whole plants	*BP/BK*	[14,45]
20	Quercetin	Twigs	*BP*	[31,45]
21	Bavachin	Whole plants	*BP*	[45]
22	Isovitexin	Leaves	*BP*	[36,45]
23	Broussofluorenone C	Whole plants	*BP*	[45]
24	Broussinol	Whole plants	*BP*	[45]
25	Sulfuretin	Whole plants	*BP*	[45]
26	Isogemichalcone C	Whole plants	*BP*	[28,45]
27	Isoliquiritigenin	Twigs	*BP*	[31,45]
28	Hesperetin	Roots	*BK*	[14]
29	Eriodictyol	Roots	*BK*	[14]
30	Chrysoeriol	Roots	*BK*	[14]
31	Kaempferol	Roots	*BK*	[14]
32	Broussonol F	Roots	*BK*	[44]
33	Broussonol G	Roots	*BK*	[40,44]
34	Broussonol H	Roots	*BK*	[44]
35	Broussonol I	Roots	*BK*	[44]
36	Broussonol K	Roots	*BK*	[44]
37	Kazinol Q	Root barks/branches and twigs	*BP/BK*	[26,40,41,43]
38	Kazinol A	Roots	*BP/BK*	[23,39,42,43,50]
39	Broussonol L	Roots	*BK*	[44]
40	Kazinol B	Roots/branches and twigs	*BP/BK*	[24,40,42,43,44]
41	Daphnegiravan D	Roots	*BK*	[44]
42	Broussonol M	Roots	*BK*	[44]
43	Broussoflavonol A	Roots/branches and twigs	*BK/BP*	[42,43,44]
44	4,2′-Dihydroxy-4′-methoxychalcone	Roots	*BK*	[44]
45	Broussonol C	Roots/leaves	*BK*	[29,44]
46	(2*S*)-2′,4′-Dihydroxy-2″-(1-hydroxy-1-methylethyl)-dihydrofuro-2,3-h flavanone	Whole plants	*BP*	[28,43]
47	(2*S*)-5,7,2′,4′-Tetrahydroxyflavanone	Whole plants	*BP*	[28,43]
48	(2*S*)-Euchrenone	Whole plants	*BP*	[28,43]
49	Broussoflavonol F	Root barks/twigs	*BP*	[24,27,30,42,49]
50	(2*S*)-Naringenin	Whole plants	*BP*	[28,43]
51	Broussoflavonol E	Root barks/twigs	*BP*	[24,38,42]
52	Broussoflavonol G	Root barks/Whole plants	*BP*	[25,42,49]
53	Broussoflavonol C	Root barks/Whole plants	*BP*	[40,43]
54	Broussoflavonol D	Whole plants	*BP*	[43]
55	4′-O-Methyldavidioside	Whole plants	*BP*	[43]
56	5,7,3′,4′-Tetrahydroxy-3-methoxy-6-geranylflavone	Whole plants/twigs	*BP*	[27,38,42]
57	Broussoflavonol B	Whole plants/branches and twigs/root barks	*BP*	[38,39,41,42]
58	5,7,3′,4′-Tetrahydroxy-6-geranylflavonol	Whole plants	*BP*	[28,43]
59	5,7,2′,4′-Tetrahydroxy-3-geranylflavone	Whole plants	*BP*	[28,43]
60	Broussochalcone A	Roots/twigs/barks	*BP*	[23,29,30,33,37,39,42,48]
61	Broussochalcone B	Roots	*BP*	[23,42,45,48]
62	(2*S*)-Abyssinone II	Whole plants	*BP*	[43]
63	(2*S*)-7,4′-Dihydroxy-3′-prenylflavan	Whole plants/twigs	*BP/BK*	[27,36,42]
64	Broussin	Branches and twigs	*BP*	[42,43]
65	Isoliquiritigenin 2′-methy ether	Whole plants	*BP*	[43]
66	1,2,4-Dihydroxy-3-(3-methylbut-2-en-1-yl)-phenyl-3-(2,4-dihydroxyphenyl)-propan-1-one	Whole plants	*BP*	[43]
67	2-{5,7-Dihydroxy-2-(4-hydroxyphenyl)-4-oxo-3,4-dihydro-2-H-chromen-8-ylamino}-pentanedioic acid	Whole plants	*BP*	[43]
68	Broussofluorenone B	Roots	*BP*	[42,48,51]
69	5,7,3′,5′-Tetrahydroxyflavanone	Twigs	*BP*	[31,43]
70	5,7,3′,4′-Tetrahydroxy-3-methoxyflavone	Twigs	*BP*	[31,43]
71	8-(1,1-Dimethylallyl)-5′-(3-methylbut-2-enyl)-3′,4′,5,7-tetrahydroxyflanvonol	Root barks/roots/twigs	*BP*	[29,38,39,42,48]
72	Kazinol E	Roots	*BP*	[42,45,48]
73	luteolin-7-*O*-*β*-D-glucopyranoside	Leaves	*BP*	[34,37,42,46]
74	Apigenin-7-*O*-*β*-D-glucoside	Leaves	*BP*	[36,43]
75	3′-*γ*-Hydroxymethyl-(*E*)-*γ*-methylallyl-2,4,2′,4′-tetrahydroxychalcone-11′-*O*-coumarate	Whole plants	*BP*	[28,43]
76	Broussoaurone A	Root barks	*BP*	[43,46]
77	Dimethoxy isogemichalcone C	Whole plants	*BP*	[43]
78	Chrysoriol-7-*O*-*β*-D-glucoside	Leaves	*BP*	[36,43]
79	Iuteoloside	Whole plants	*BP*	[43]
80	3,4-Dihydroxyisolonchocarpin	Roots	*BP*	[42,45,48]
81	(2*S*)-2′,4′-Dihydroxy-2″(1-hydroxy-1-methylethyl)-dihydrofurano-2,3-h-flavanone	Whole plants	*BP*	[43]
82	5,7,3′,4′-Tetrahydroxy-3-methoxy-8-geranylflavone	Barks	*BP*	[33,37,42]
83	5,7,3′,4′-Tetrahydroxy-3-methoxy-8,5′-diprenylflavone	Barks/branches and twigs	*BP*	[33,41,42]
84	Fipsotwin	Branches and twigs	*BP*	[42]
85	Kazinol N	Branches and twigs	*BP*	[42]
86	Kazinol M	Branches and twigs	*BP*	[42]
87	Threo-dadahol B	Branches and twigs	*BP*	[42]
88	Threo-dadahol A	Branches and twigs	*BP*	[42]
89	Broussoflavonol H	Root barks	*BP*	[41]
90	Broussoflavonol I	Root barks	*BP*	[41]
91	Broussoflavonol J	Root barks	*BP*	[41]
92	Broussoflavonol K	Root barks	*BP*	[41]
93	Glycyrrhiza flavonol A	Root barks	*BP*	[41]
94	Isolicofavonol	Root barks	*BP*	[41]
95	Broussoflavonol F	Root barks	*BP*	[41]
96	Broussoflavonol B	Root barks	*BP*	[41]
97	(2*R*)-7,3′,4′-Trihydroxy-6-prenylflavanone	Root barks	*BP*	[40]
98	Broussochalcone C	Root barks	*BP*	[40]
99	Broussoflavanonol A	Root barks	*BP*	[40]
100	Broussonol D	Root barks/leaves/twigs	*BP/BK*	[28,38,39]
101	Daphnegiravan H	Root barks	*BP*	[40]
102	(-)-(2*S*)-Kazinol I	Root barks	*BP*	[40]
103	Broupapyrin A	Twigs	*BP*	[39]
104	8-Prenylquercetin-3-methyl ether	Twigs	*BP*	[39]
105	4,2′,4′-Trihydroxychalcone	Twigs	*BP*	[39]
106	Butein	Twigs	*BP*	[39]
107	Broussonol E	Twigs	*BP*	[39]
108	7,4′-Dihydroxy-3′-prenylflavan	Whole plants	*BP*	[38]
109	7,3′-Dihydroxy-4′-methoxyflavan	Twigs	*BP/BK*	[37,38]
110	3′-(3-Methylbut-2-enyl)-3′,4′,7-trihydroxyflavane	Twigs/roots	*BP*	[29,30,37,42,48,52]
111	Brossoflurenone A	Roots	*BP*	[38,47]
112	Brossoflurenone B	Roots	*BP*	[38,47]
113	Apigenin-7-*O*-*β*-D-glucopyranoside	Leaves	*BP*	[38,48]
114	Apigenin-6-C-*β*-D-glucopyranside	Leaves	*BP*	[34,37,42]
115	Bropapyrifero	Whole plants	*BP*	[17]
116	(−)-Broukazinol A	Twigs	*BK*	[37]
117	(+)-Broukazinol A	Twigs	*BK*	[37]
118	(2*R*)-7,4′-Dihydroxy-3′-prenylflavan	Twigs	*BK*	[37]
119	(2*R*)-7,4′-Dihydroxyflavan(tupichinol C)	Twigs/root barks/stem barks	*BK*	[26,32,36]
120	(2*S*)-7,4′-Dihydroxyflavan(demethylbroussin)	Twigs/root barks/stem barks	*BK*	[26,32,36]
121	Broussoside F	Twigs	*BK*	[37]
122	(2*S*)-7,3′-Dimethoxy-4′-hydroxyflavan	Twigs	*BK*	[37]
123	Kazinol I	Twigs/root barks	*BK*	[37,49]
124	Tupichinol C	Root barks	*BK*	[49,50]
125	Kazinol U	Root barks	*BK*	[49,50]
126	3,5,7,4′-Tetrahydroxy-3′-(2-hydroxy-3-methylbut-3-enyl) flavone	Twigs	*BP*	[31,51]
127	Luteoloside	Leaves	*BP*	[36]
128	Broussoflavonol B	Barks	*BP*	[34]
129	3′,7-Dihydroxy-4′-methoxyflavan	Stem barks	*BK*	[33]
130	3,7-Dihydroxy-4′-methoxyflavone	Stem barks	*BK*	[33]
131	3,7,3′-Trihydroxy-4′-methoxyflavone	Stem barks	*BK*	[33]
132	(+) − (2*R*) Kazinol I	Whole plants	*BK*	[50]
133	Apigenin-7-*O*--glucopyranoside	Leaves	*BP*	[32]
134	Amentoflavone	Leaves	*BP*	[32]
135	3,3′,4′,5,7-Pentahydroxyflavone	Roots	*BP*	[30]
136	Broussonol A	Leaves	*BK*	[29]
137	Broussonol B	Leaves	*BK*	[29]
138	Broussonol E	Leaves	*BK*	[29]
139	2,4,2′,4′-Tetrahydroxy-3′-prenylchalcone	Whole plants	*BP*	[28]
140	(2*S*)-2′,4′-Dihydroxy-7-methoxy-8-prenylflavan	Whole plants	*BP*	[28]
141	Australone A	Root barks	*BP*	[52]
142	Cyclomorusin	Whole plants	*BP*	[26]
143	Cycloartomunin	Whole plants	*BP*	[26]
144	Dihydroisocycloartomunin	Whole plants	*BP*	[26]

**Table 2:** Penylpropanoids isolated from *Broussonetia* species.

Number	Compounds	Parts	Source	References
145	Marmesin	Whole plants	*BP*	[45]
146	Graveolone	Whole plants	*BP*	[45]
147	Sesquineolignan	Whole plants	*BP*	[45]
148	Dihydrosyringin	Leaves	*BP*	[36,45]
149	Coniferyl alcohol	Fruits	*BP*	[43,45,54]
150	Ferulic acid	Fruits	*BP*	[45,54]
151	*p*-Coumaraldehyde	Fruits	*BP*	[45,54]
152	Liriodendrin	Leaves	*BP*	[35,45]
153	Dihydro-coniferyl alcohol	Whole plants	*BP*	[45]
154	Chushizisin G	Fruits	*BP*	[45,53]
155	Chushizisin H	Fruits	*BP*	[45,53]
156	Pinoresinol	Roots	*BK*	[14]
157	3′-Hydroxymarmesin-1′-*O*-*β*-glucopyranosyl	Roots	*BK*	[14]
158	Marmesinin	Roots	*BK*	[14]
159	Syringaresinol-4-*O*-*β*-D-glucopyranoside	Roots	*BP/BK*	[14,38]
160	Rutaretin methylether	Roots	*BK*	[44]
161	Fipsomin	Roots	*BK*	[44]
162	(*S*)-Marmesin	Branches and twigs	*BP*	[42]
163	(+)-Marmesin	Whole plants	*BP*	[43]
164	(+)-Pinoresinol-4′-*O*-*β*-D-glucopyranosyl-4″-*O*-*β*-D-apiofuranoside	Leaves	*BP*	[35,43]
165	Syringaresinol-4′-*O*-*β*-D-glucoside	Leaves	*BP*	[36,43]
166	Pinoresinol-4′-*O*-*β*-D-glucopyranoside	Leaves	*BP*	[36,43]
167	(*S*)-8-Methoxymarmesin	Branches and twigs	*BP*	[42,43]
168	7,8-Dihydroxy-6-(3-methylbut-2-en-1yl)-2H-chromen-2-one	Root barks	*BP*	[41,43]
169	Broussocoumarin A	Root barks	*BP*	[41,43]
170	Cissyringin	Fruits	*BP*	[43,54]
171	Cisconiferin	Fruits	*BP*	[43,54]
172	Chushizisin A	Fruits	*BP*	[43,53]
173	Chushizisin B	Fruits	*BP*	[43,53]
174	Chushizisin C	Fruits	*BP*	[43,53]
175	Chushizisin D	Fruits	*BP*	[43,53]
176	Chushizisin E	Fruits	*BP*	[43,53]
177	Chushizisin F	Fruits	*BP*	[43,53]
178	*p*-Coumaric acid	Leaves	*BP*	[36,43]
179	Threo-1-(4-hydroxy-3-methoxyphenyl)-2-{4-(*E*)-3-hydroxy-1-propenyl-2-methoxyphenoxy}-1,3-propanediol	Fruits	*BP*	[43,53]
180	Erythro-1-(4-hydroxyphenyl) glycerol	Fruits	*BP*	[43,54]
181	Threo-1-(4-hydroxyphenyl) glycerol	Fruits	*BP*	[43,54]
182	Erythro-1-(4-hydroxy-3-methoxyphenyl)-2-{4-(*E*)-3-hydroxy-1-propenyl-2-methoxy-phenoxy}-1,3-propanediol	Fruits	*BP*	[43,53]
183	3-2-(4-Hydroxyphenyl)-3-hydroxymethyl-2,3-dihydro-1-benzofuran-5-ylpropan-1-ol	Fruits	*BP*	[43,53]
184	Chushizisin I	Fruits	*BP*	[43,53]
185	6,7-Dimethoxycoumarin	Whole plants	*BP*	[38]
186	(+)-(2′*S*,3′*R*)-3-Hydroxyl marmesin	Whole plants	*BP*	[38]
187	Iariciresinol-9-*O*-*β*-D-glucopyranoside	Whole plants	*BP*	[38]
188	3,4′,5′-Trihy- droxy-5-methoxy-6H-benzo [c] chromen-6-one	Whole plants	*BP*	[38]
189	Alternariol-4′-*O*-methyl ether	Whole plants	*BP*	[38]
190	Alternariol-5-*O*-methyl ether	Whole plants	*BP*	[38]
191	Alternariol	Whole plants	*BP*	[38]
192	Alternuene	Whole plants	*BP*	[38]
193	(7*S*,7′*S*,7″*R*,8*R*,8′*R*,8″*S*)-3′-Methoxy-4,4″,9″- trihydroxy- 4′,7″,7,9′,7′,9-triepoxy-5′,8″,8,8″-sesquineolignan	Whole plants	*BP*	[51]
194	Dihydroconiferyl alcohol	Fruits	*BP*	[54]

**Table 3:** Polyphenols isolated from *Broussonetia* species.

Number	Compounds	Parts	Source	References
195	Broussonin A	Twigs/stem barks/root barks/whole plants	*BP/BK*	[28,33,37,40,45,49,50]
196	Broussonin B	Twigs/stem barks/root barks	*BP/BK*	[33,37,40,45]
197	Resveratrol	Whole plants	*BP*	[45]
198	Moracin N	Whole plants	*BP*	[28,43,45]
199	Demethylmoracin I	Whole plants	*BP*	[28,45]
200	Mulberrofuran G	Whole plants	*BP*	[45]
201	Curculigoside C	Fruits	*BP*	[45,54]
202	Protocatechuic acid	Whole plants	*BP*	[45]
203	Kazinol K	Roots/root barks	*BK*	[27,44]
204	Kazinol F	Twigs/leaves/root barks	*BP/BK*	[29,37,40,43,55]
205	1-(2,4-Dihydroxyphenyl)-3-(4-hydroxyphenyl)-propane	Whole plants	*BP*	[28,43]
206	1-(4-Hydroxy-2-methoxyphenyl)-3-(4-hydroxy-3-prenylphenyl)-propane	Twigs/whole plants	*BP/BK*	[28,37,43]
207	Broussonin C	Root barks	*BP/BK*	[43,55]
208	Moracin I	Whole plants	*BP*	[43]
209	Moracin D	Whole plants	*BP*	[43]
210	Broussonin F	Twigs	*BP/BK*	[37,43]
211	Moracin M	Whole plants	*BP*	[43]
212	Broussonin E	Roots	*BP/BK*	[43,44]
213	3,5,4′-Trihydroxy-bibenzyl-3-*O*-*β*-D-glucoside	Leaves	*BP*	[35,43]
214	Broussoside D	Leaves	*BP*	[36,43]
215	Broussofluorenone A	Roots	*BP*	[43,56]
216	Kazinol V	Roots/root barks	*BP/BK*	[40,44]
217	Kazinol J	Root barks/leaves	*BP/BK*	[29,40]
218	Kazinol W	Root barks	*BP*	[40]
219	Altertoxin IV	Whole plants	*BP*	[38]
220	Altertoxin I	Whole plants	*BP*	[38]
221	Broukazinol B	Twigs	*BK*	[37]
222	Broukazinol C	Twigs	*BK*	[37]
223	1-(2,4-Dihydroxy-3-prenylphenyl)-3-(4-hydroxyphenyl)-propane	Twigs/whole plants	*BK/BP*	[28,37]
224	Kazinol S	Twigs/root barks	*BK*	[37,55]
225	Kazinol C	Root barks/twigs	*BP/BK*	[31,49,55]
226	Kazinol D	Root barks/twigs	*BP/BK*	[27,31,49,55]
227	(7′*R*,8′*S*) -3-Methoxy-4′,9,9″-trihydroxy-4,7′-epoxy-5,8′-neolignan	Whole plants	*BP*	[51]
228	(7*R*,8*S*,8′*R*)-7″,8″-Threo-3′-methoxy-7′-oxo-4,4″,7″,9,9″-pentahydroxy-4′,8″: 7,9′-bis-epoxy-8,8′-sesquineolignan	Fruits	*BP*	[51,54]
229	Broussonone A	Stem barks/roots	*BP/BK*	[33,57]
230	3,4-Dihydroxybenzoic acid	Fruits	*BP*	[54]
231	Kazinol T	Root barks	*BK*	[55]
232	Albanol A	Whole plants	*BP*	[28]

**Table 4:** Alkaloids isolated from *Broussonetia* species.

Number	Compounds	Parts	Source	References
233	Liriodenine	Fruits	*BP*	[45,67]
234	Isoterihanine	Whole plant	*BP*	[45]
235	Chelerythrine	Whole plants	*BP*	[45]
236	Oxyavicine	Fruits	*BP*	[45,67]
237	Broussonpapyrine	Fruits	*BP*	[45,67]
238	Nitidine	Fruits	*BP*	[43,67]
239	2′-Deoxyuridine	Whole plants	*BP*	[43]
240	2′-Deoxyadenosine	Whole plants	*BP*	[43]
241	Thymidine	Whole plants	*BP*	[43]
242	N-Norchelerythrine	Fruits	*BP*	[66]
243	Dihydrosanguinarine	Fruits	*BP*	[66]
244	Broussonetine R	Branches	*BK*	[65]
245	Broussonetine S	Branches	*BK*	[65]
246	Broussonetine T	Branches	*BK*	[65]
247	Broussonetine U	Branches	*BK*	[65]
248	Broussonetine V	Branches	*BK*	[65]
249	Broussonetine M	Branches	*BK*	[64]
250	Broussonetine O	Branches	*BK*	[64]
251	Broussonetine P	Branches	*BK*	[64]
252	Broussonetine Q	Branches	*BK*	[64]
253	Broussonetine A	Branches	*BK*	[59,68]
254	Broussonetinine A	Branches	*BK*	[59,68]
255	Broussonetine B	Branches	*BK*	[59,68]
256	Broussonetinine B	Branches	*BK*	[59,68]
257	Broussonetine C	Branches	*BK*	[58,68]
258	Broussonetine E	Branches	*BK*	[59,68]
259	Broussonetine D	Branches	*BK*	[58,68]
260	Broussonetine F	Branches	*BK*	[59,68]
261	Broussonetine G	Branches	*BK*	[61,68]
262	Broussonetine H	Branches	*BK*	[61,68]
263	Broussonetine I	Branches	*BK*	[60,68]
264	Broussonetine J	Branches	*BK*	[60,68]
265	Broussonetine K	Branches	*BK*	[63,68]
266	Broussonetine L	Branches	*BK*	[63,68]
267	Broussonetine N	Branches	*BK*	[62]

**Table 5:** Terpenoids isolated from *Broussonetia* species.

Number	Compounds	Parts	Source	References
268	Lupeol acetate	Whole plants	*BP*	[38]
269	Augustic acid	Whole plants	*BP*	[38]
270	3*β*-acetoxy–tirucalla-7-en-24*S*,25-diol	Barks	*BP*	[38,70]
271	Lupeol	Barks	*BP*	[70]
272	*β*-Amyrin	Barks	*BP*	[70]
273	*α*-Amyrin acetate	Barks	*BP*	[70]
274	(3*β*)-3-(acetyloxy)-eupha-7,25-dien-24-one	Barks	*BP*	[38,69]
275	(3*β*,24*R*)-3-(acetyloxy)–eupha-7,25-dien-24-ol	Barks	*BP*	[38,69]
276	(3*β*,24*S*)-eupha-7,25-diene-3,24-diol	Barks	*BP*	[38,69]
277	(3*β*,24*R*)-Eupha-7,25-diene3,24-diol	Barks	*BP*	[69]
278	Taraxerol acetate	Leaves	*BP*	[32]
279	Broussonetone A	Leaves	*BP*	[32,51]
280	Broussonetone B	Leaves	*BP*	[32,51]
281	Broussonetone C	Leaves	*BP*	[32,51]
282	Oleanolic acid	Root barks	*BP/BK*	[27,38]
283	Squalene	Fruits/root barks/leaves	*BP/BL*	[25,45,53,71]
284	Butyrospermol acetate	Whole plants	*BP*	[24]

**Table 6:** Steroids isolated from *Broussonetia* species.

Number	Compounds	Parts	Source	References
285	Fucosterol	Whole plants	*BP*	[45]
286	Ergosterol peroxide	Whole plants	*BP*	[45]
287	*β*-Sitosterol	Whole plants	*BP*	[43]
288	*β*-Daucosterol	Whole plants	*BP*	[43]
289	Ergosta-4,6,8,22-tetraen-3-one	Whole plants	*BP*	[43]

**Table 7:** Other compounds isolated from *Broussonetia* species.

Number	Compounds	Parts	Source	References
290	Arbutin	Whole plants	*BP*	[45]
291	Broussoside B	Whole plants	*BP*	[45]
292	D-Galacitol	Whole plants	*BP*	[43]
293	Daucosterol palmitate	Whole plants	*BP*	[38]
294	Palmitic acid ethyl ester	Whole plants	*BP*	[38]
295	Palmitic acid	Whole plants	*BP*	[38]
296	Linoleic acid	Whole plants	*BP*	[38]
297	9-Octadecenoic acid	Whole plants	*BP*	[38]
298	8,11-Octadecadienoic acid	Whole plants	*BP*	[38]
299	*α*-Monopalmitin	Whole plants	*BP*	[38]
300	Monoheptadecanoin	Whole plants	*BP*	[38]
301	Heptadecanoic acid	Whole plants	*BP*	[38]
302	Phytol	Whole plants/leaves	*BP/BL*	[38,71]
303	Physcion	Whole plants	*BP*	[38]
304	Altersolanol A	Whole plants	*BP*	[38]
305	Altersolanol C	Whole plants	*BP*	[38]
306	*δ*-Tocopherol	Whole plants	*BP*	[38]
307	(4*R*,5*S*,10*S*)-8,9,10-Trihydroxy-4-[3′-methoxy-4′-hydroxyphenyl]-1,6-dioxaspiro [4,5] decan-2-one	Whole plants	*BP*	[38]
308	4-Hydroxyacetophenone	Whole plants	*BP*	[38]
309	Erythro-1-(4-hydroxyphenyl)-2-{4-[(*E*)-3-hydroxy-1-propenyl]-2-methoxyphenoxy}-1,3-propanediol	Whole plants	*BP*	[51]
310	Threo-1-(4-hydroxyphenyl)-2-{4-[(*E*)-3-hydroxy1-propenyl]-2-methoxyphenoxy}-1,3-propanediol	Whole plants	*BP*	[51]
311	threo-1-(4-hydroxyphenyl)-2-[4-(3-hydroxy-1-propyl)-2-methoxyphenoxy]-1,3-propanediol	Whole plants	*BP*	[51]
312	erythro-1-(4-hydroxyphenyl)-2-[4-(3-hydroxy-1-propyl)-2-methoxyphenoxy]-1,3-propanediol	Whole plants	*BP*	[51]
313	(7′*R*,8′*S*)-3-Methoxy-7-oxo-4′,9,9″-trihydroxy-4,7′-epoxy-5,8′-neolignan	Whole plants	*BP*	[51]
314	(7′*R*,8′*S*)-3-Methoxy-4′,9,9″-trihydroxy-4,7′-epoxy-5,8′-neolignan	Whole plants	*BP*	[51]
315	Benzyl benzoate-2,6-di-*O*-*β*-D-glucopyranoside	Whole plants	*BP*	[51]
316	Broussoside A	Twigs/leaves	*BP/BK*	[36,37,43]
317	Broussoside C	Leaves	*BP*	[36,43]
318	Broussoside E	Leaves	*BP*	[36,43]
319	Flacourtin	Leaves	*BP*	[36,45]
320	Poliothyrsoside	Leaves	*BP*	[36,45]
321	Adenosine	Seeds	*BP*	[73]
322	Chushizilactam A	Seeds	*BP*	[73]
323	Arbutine	Fruits	*BP*	[54]
324	4-Hydroxybenzaldehyde	Fruits	*BP*	[43,45,54]
325	Curculigoside I	Fruits	*BP*	[43,54]
326	2-(4-Hydroxyphenyl) propane-1,3-diol-1-*O*-*β*-D-glucopyranoside	Fruits	*BP*	[43,54]
327	(2*R*,3*R*,5*R*,6*S*,9*R*)-3-Hydroxy-5,6-epoxyb-ionol-2-*O*-*β*-D-glucopyranoside	Leaves	*BP*	[43,72]
328	(2*R*,3*R*,5*R*,6*S*,9*R*)-3-Hydroxyl-5,6-epoxy-acety-b-ionol-2-*O*-*β*-D-glucopyranoside	Leaves	*BP*	[72]
329	Lignoceric acid	Root barks	*BP*	[25,45]
330	Octacosan-1-ol	Root barks	*BP*	[25,45]
331	4′-Hydroxycis-cinnamic acid octacosyl ester	Root barks	*BP*	[25]
332	Erythrinasinate	Root barks	*BP*	[25]
333	1,2,3-Propanetriol, monoacetate	Leaves	*BL*	[71]
334	1,2,3-Propanetriol, diacetate	Leaves	*BL*	[71]
335	Hexadecanoic acid, ethyl ester	Leaves	*BL*	[71]
336	9,12,15-Octadecatrienoic acid, methyl ester, (Z, Z, Z)-	Leaves	*BL*	[71]
337	9,12,15-Octatrienoic acid, ethyl ester, (Z, Z, Z)-	Leaves	*BL*	[71]
338	3,7,11,15-Tetramethyl-2-hexadecen-1-ol	Leaves	*BL*	[71]

## Data Availability

Not applicable.

## Supplementary Materials

The following are available online at https://www.mdpi.com/article/10.3390/molecules27165344/s1, Figure S1: Chemical structures of the Flavonoids in Broussonetia species, Figure S2: Chemical structures of the Penylpropanoids in Broussonetia species, Figure S3: Chemical structures of the Polyphenols in Broussonetia species, Figure S4: Chemical structures of the Alkaloids in Broussonetia species, Figure S5: Chemical structures of the Terpenoids in Broussonetia species, Figure S6: Chemical structures of the Steroids in Broussonetia species, Figure S7: Chemical structures of the Others in Broussonetia species.

## Abbreviations

CALCBP	Chlorogenic acid-like compounds extracted from BP
MS	Mass Spectrum
NMR	Nuclear Magnetic Resonance Spectrometer
EC	Esophagus cancer
NSCLC	Non-small cell lung cancer cell
ERK	Extracellular signal-regulated kinase
AMPK	Adenosine 5-monophosphate [134]-activated protein kinase
HepG	Human hepatoma carcinoma
CAA	Cellular antioxidant activity
IC_50_	Half maximal inhibitory concentration
XOD	Xanthine oxidase
TFC	Determination of total flavonoid content
TPC	Determination of total phenolic contents
MTT	3-(4,5)-dimethylthiahiazo (-z-y1)-3,5-di- phenytetrazoliumromide
TBARS	Thiobarbituric acid-reactive substance
PMA	Phorbol 12-myristate 13-acetate
PHA	Phytohemagglutinin
iNOS	Inducible nitric oxide synthas
LPS	Lipopolysaccharide
PA-	Palmitate-
STZ	Streptozotocin
HFD	High fat diet
MLDS	Multiple low-dose streptozotocin
ALT	Alanine aminotransferase
OLETF	Otsuka Long-Evans Tokushima fatty
MIC	Minimum inhibitory concentration
APAP	Acetaminophen
hHFDP	Human hair follicle dermal papilla
BChE	Butylcholinesterase
hAChE	Human acetylcholinesterase
OVA	Ovalbumin
BW	Body weight
ADG	Average daily gain
DMI	Dry matter intake
FCR	Feed conversion ratio
MDA	Malondialdehyde
SOD	Superoxide dismutase
TAC	Total antioxidant capacity
PUFA	Polyunsaturated fatty acids
DHA	Docosahexaenoic acid
LPS	Lipopolysaccharide
PTP1B	Tyrosine phosphatase 1B protein
MAPK	Mitogen-activated protein kinase
STAT	Signal transducer and activator of transcription
JAK	Janus kinase
AD	Atopic dermatitis
T24	Human bladder cancer cells
DPPH	1,1′-diphenyl-2-picryl-hydrazyl radical
ABTS	2,2′-azino-bis (3-ethylbenzothiazoline-6-sulfonic acid
FRAP	Ferric reducing activity power assay
TEAC	Trolox equivalent antioxidant capacity
TNF-alpha	Tumor necrosis factor-alpha
IFN-gamma	Interferongamma
TARC/CCL17	Thymus-and-activation regulated chemokine
MDC/CCL22	Macrophagederived chemokine
RANTES/CCL5	Regulated-onactivation-normal T cell-expressed-and-secreted chemokine
IL-4	Interleukin-4
IgE	Plasma immunoglobulin E
RINm5F cells	Rat pancreatic β-cell line
HFD	Ten-week-high fat diet
