# Supplementary material for: The Genus Broussonetia: An Updated Review of Phytochemistry, Pharmacology and Applications
Source: Molecules. 2022 Aug 22;27(16):5344. doi: 10.3390/molecules27165344 (PMC9414938; doi:10.3390/molecules27165344)
Supplement: Supplementary file 1 [file molecules-27-05344-s001.zip › molecules-1831551-supplementary.pdf]

Figure S1. Chemical structures of the Flavonoids in *Broussonetia* species.

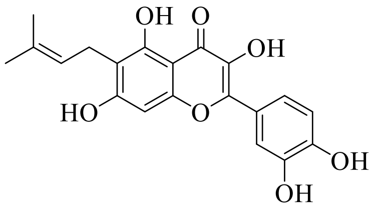

1

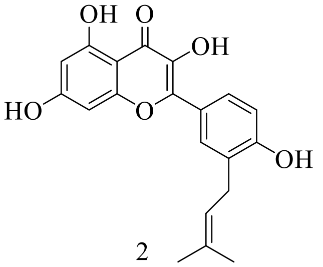

2

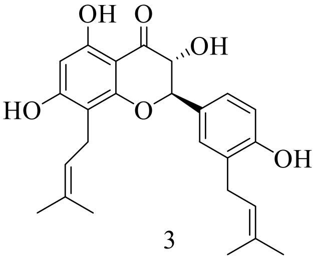

3

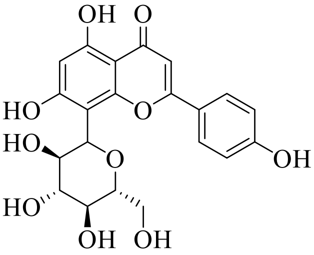

4

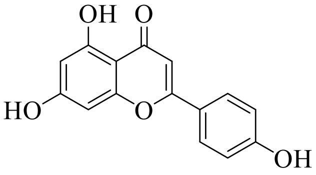

5

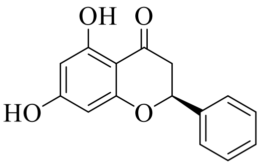

6

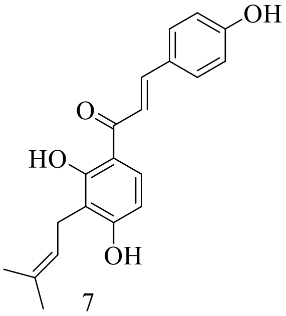

7

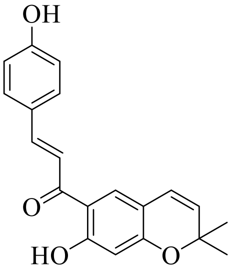

8

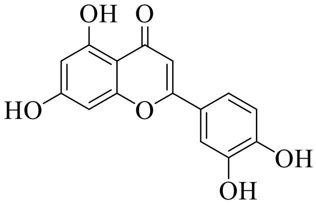

9

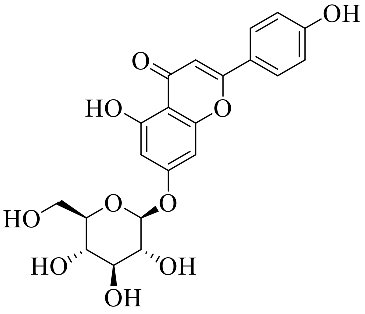

10

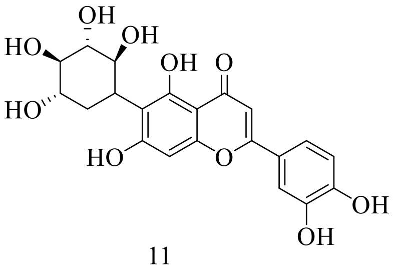

11

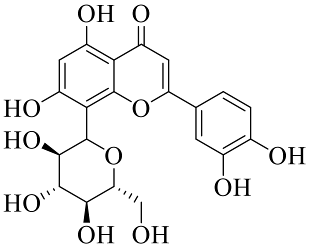

12

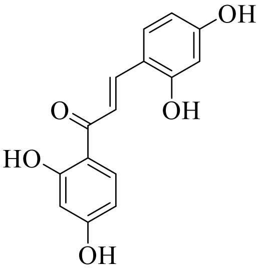

13

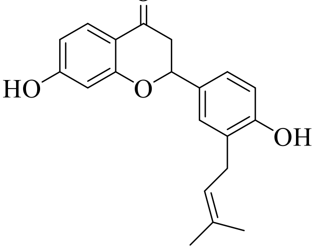

14

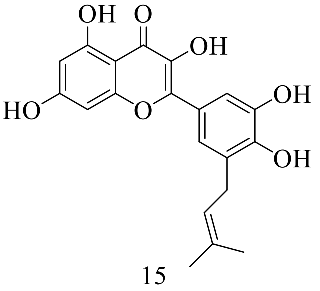

15

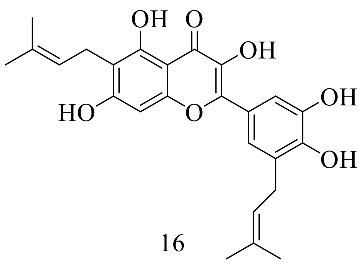

16

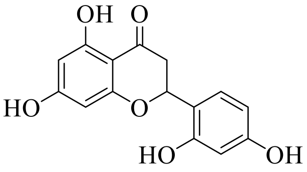

17

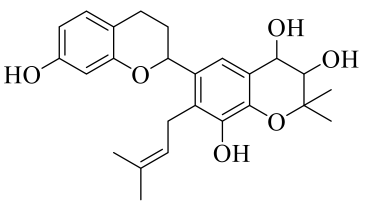

18

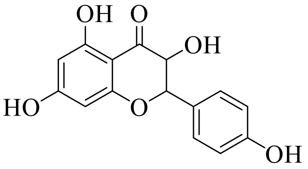

19

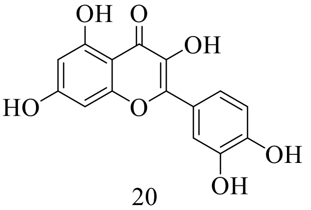

20

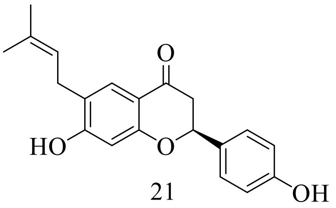

21

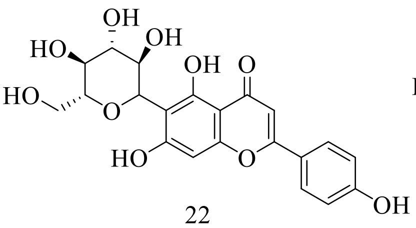

22

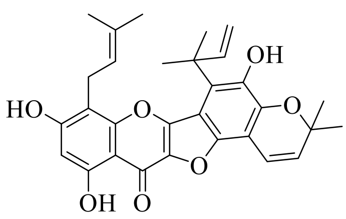

23

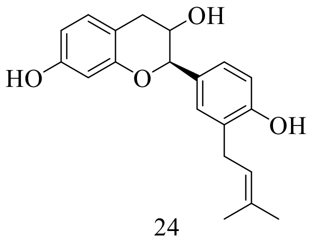

24

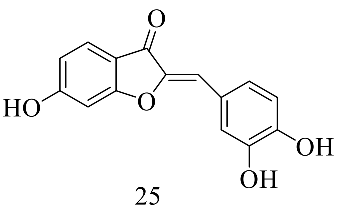

25

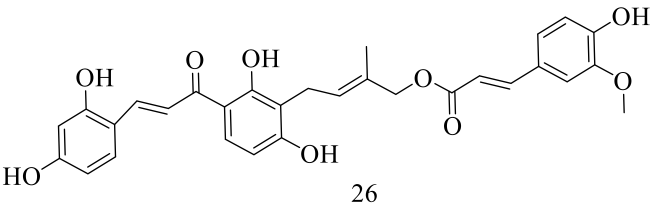

26

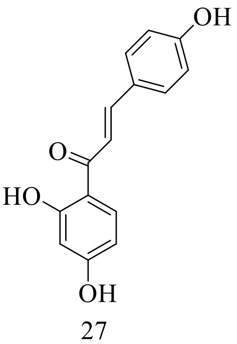

27

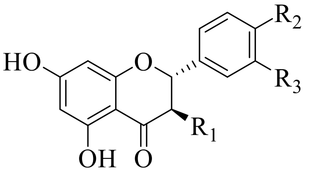

R1	R2	R3	
H2	OCH_3	OH	28
H2	OH	OH	29

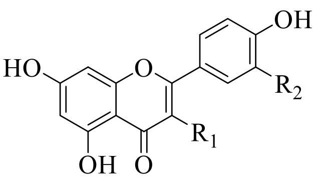

R1	R2	
H	OCH_3	30
OH	H	31

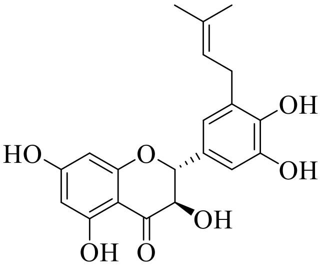

32

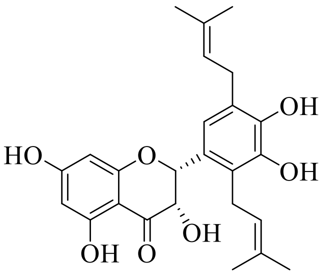

33

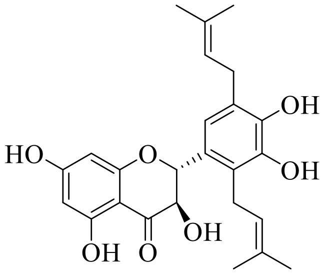

34

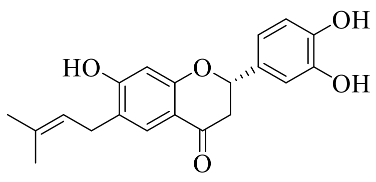

35

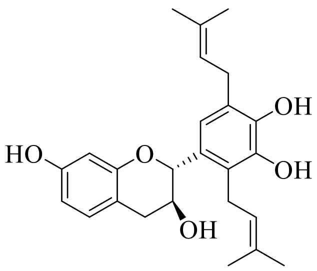

36

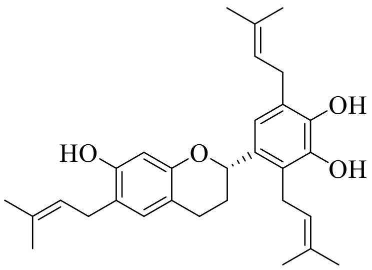

37

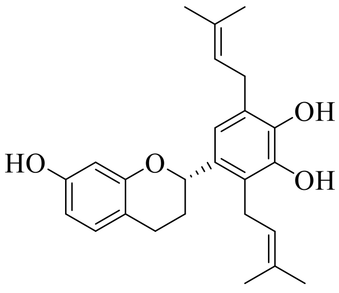

38

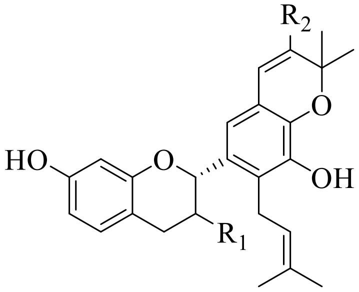

R_1	R_2	
OH	H	39
H	H	40
H	OCH_3	41

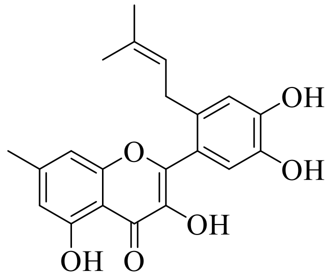

42

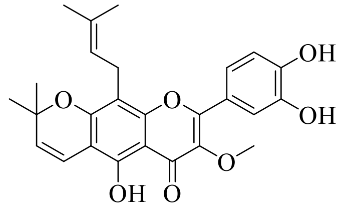

43

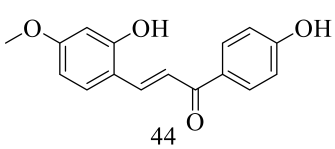

44

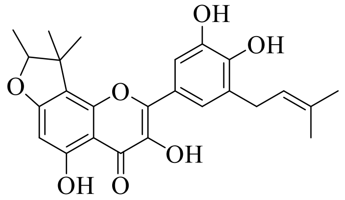

45

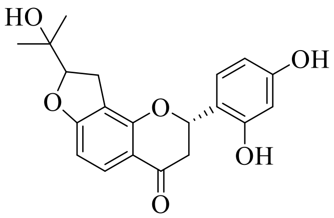

46

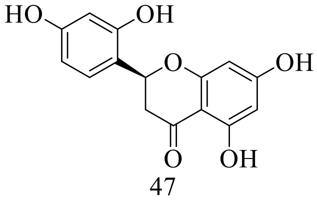

47

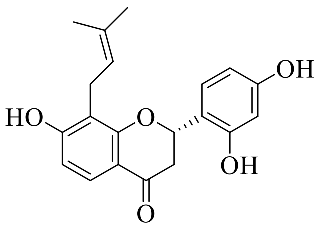

48

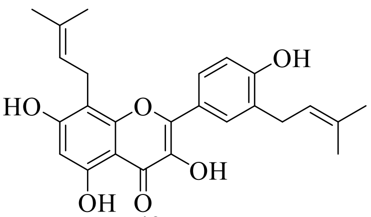
49

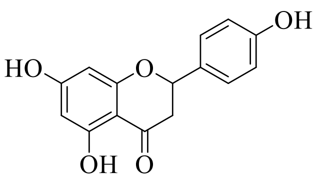
50

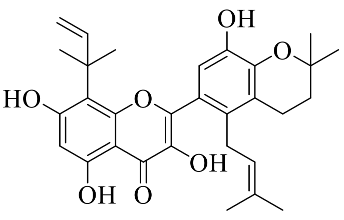
51

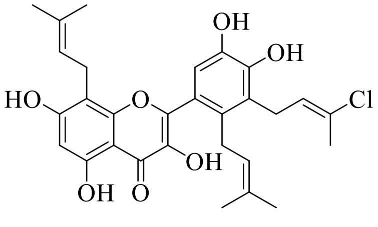
52

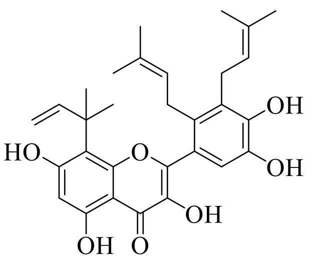
53

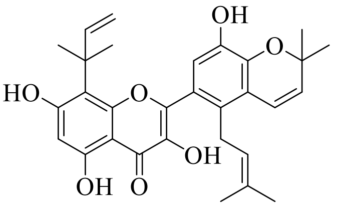
54

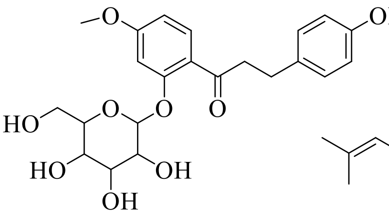
55

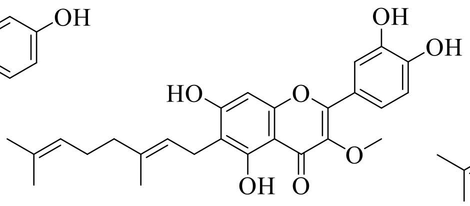
56

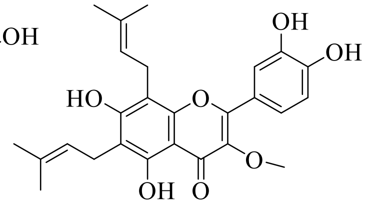
57

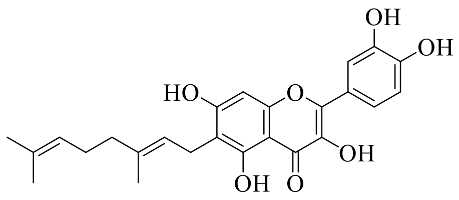
58

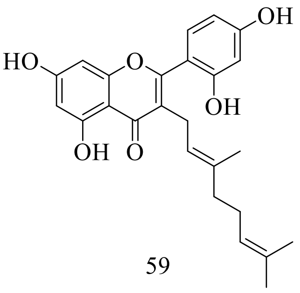
59

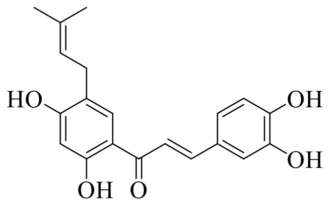
60

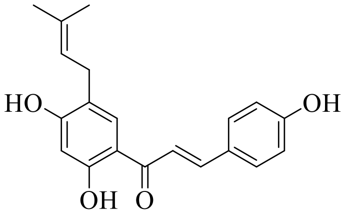
61

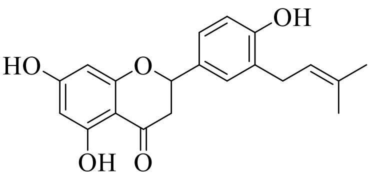
62

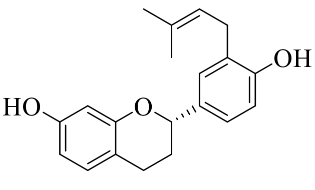
63

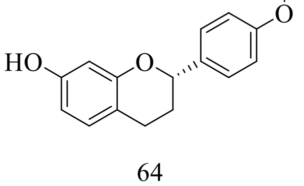
64

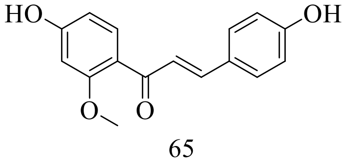
65

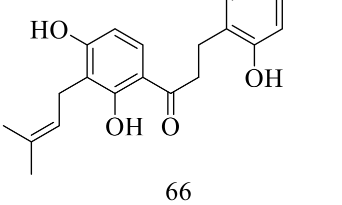
66

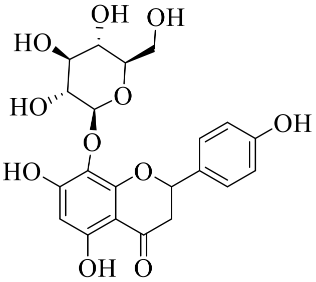

67

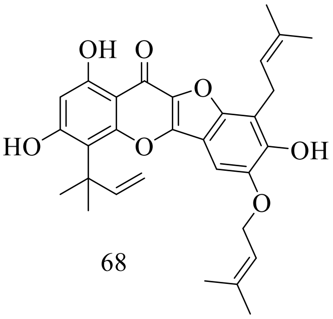

68

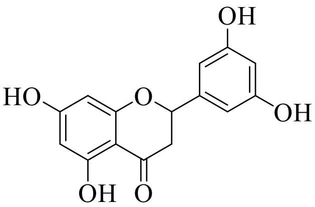

69

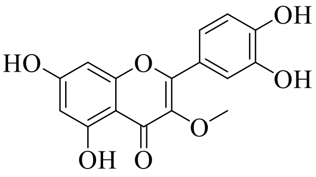

70

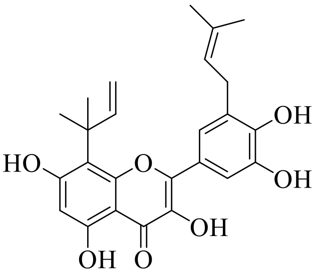

71

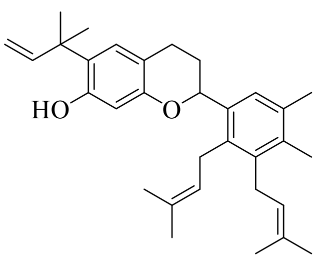

72

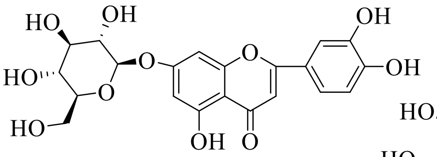

73

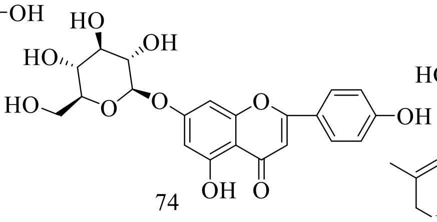

74

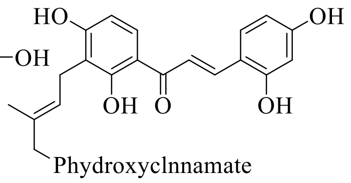

75

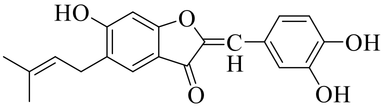

76

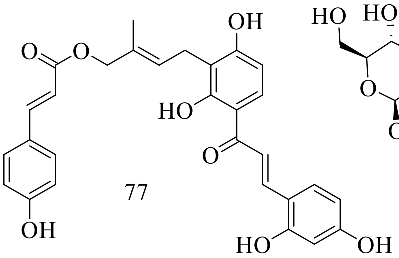

77

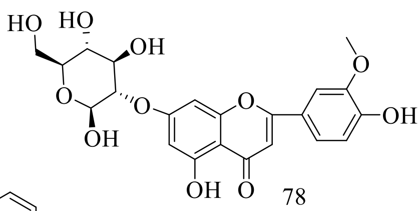

78

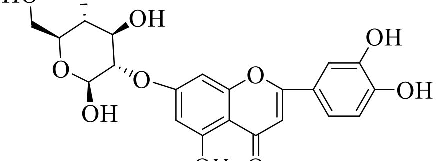

79

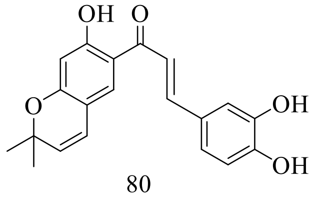

80

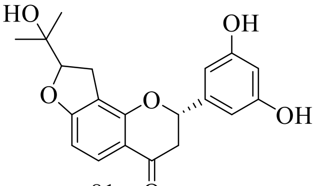

81

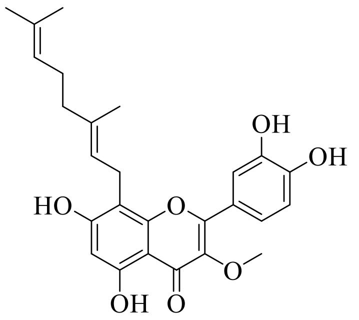

82

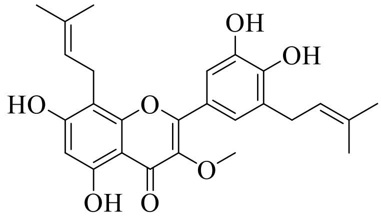

83

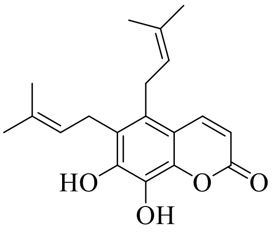

84

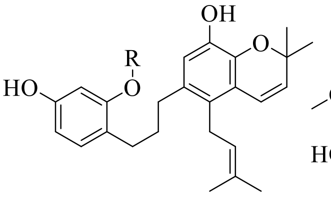

R=CH_3 85

R=H 86

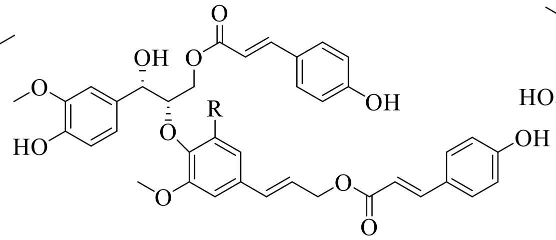

R=H 87

R=OCH_3 88

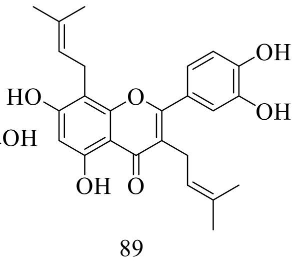

89

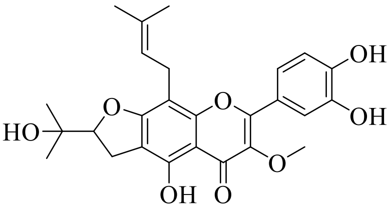

90

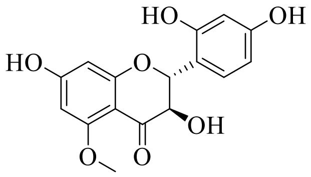

91

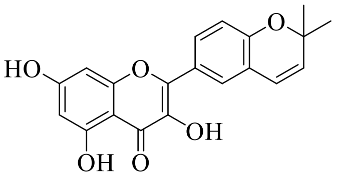

92

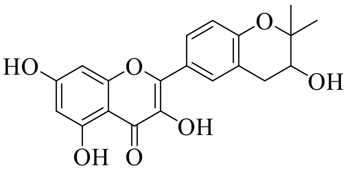

93

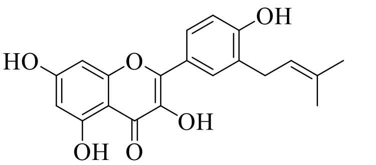

94

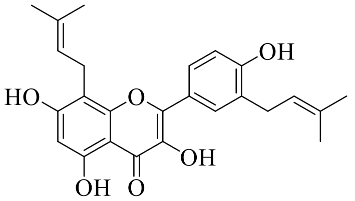

95

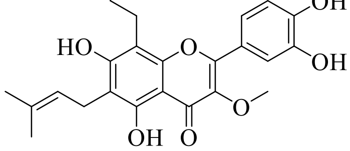

96

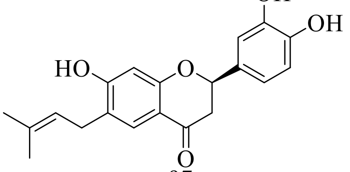

97

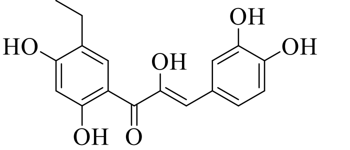

98

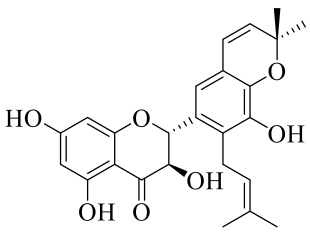
99

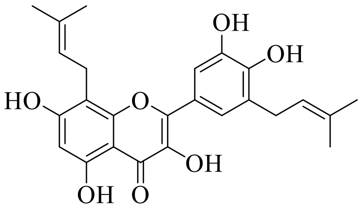
100

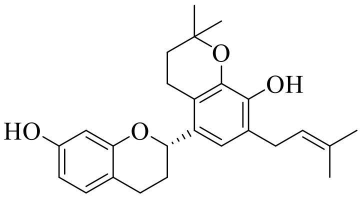
101

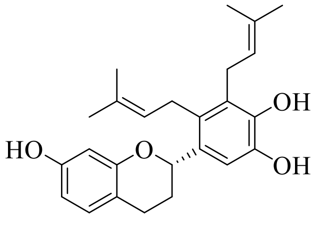
102

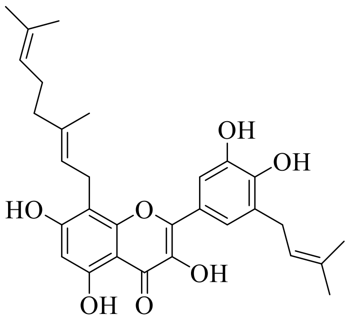
103

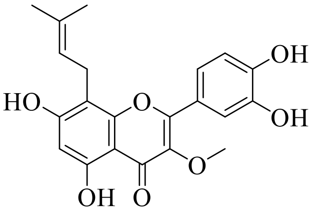
104

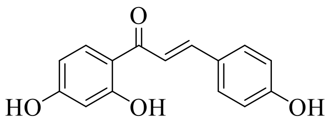
105

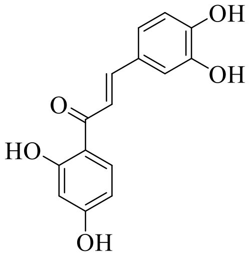
106

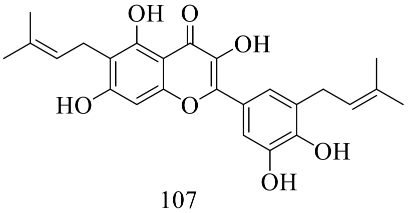
107

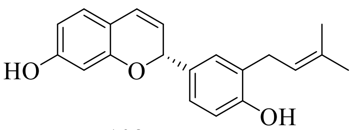
108

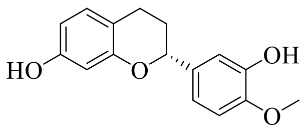
109

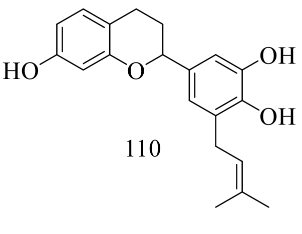
110

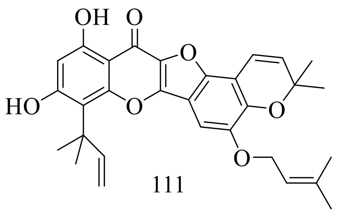
111

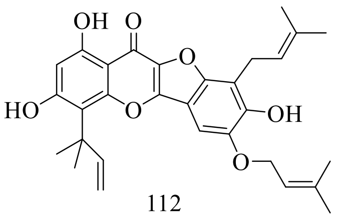
112

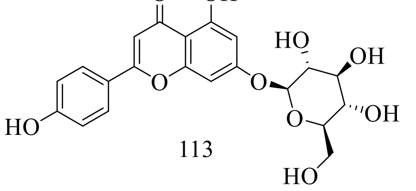
113

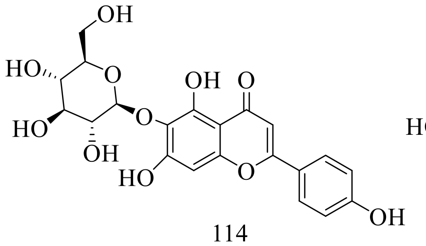

114

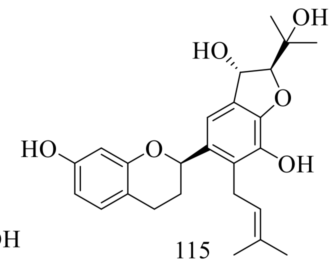

115

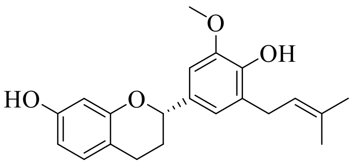

116

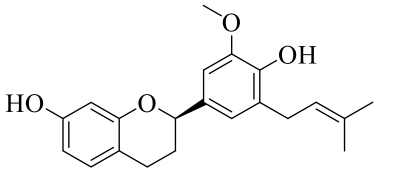

117

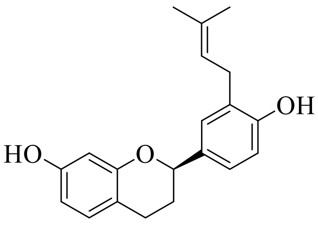

118

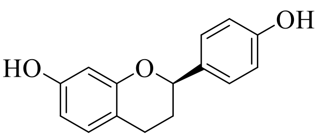

119

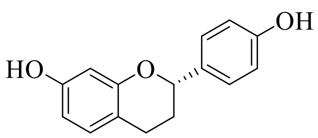

120

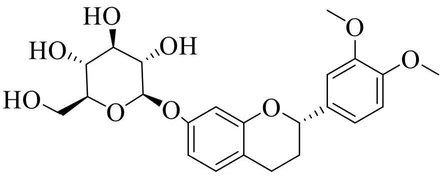

121

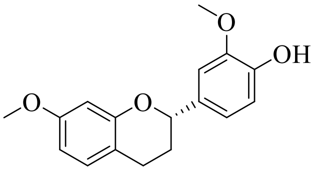

122

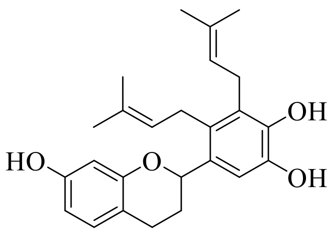

123

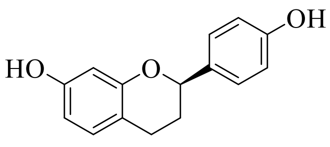

124

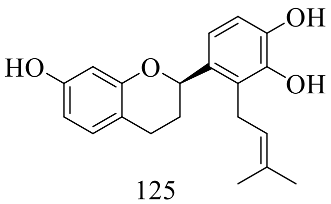

125

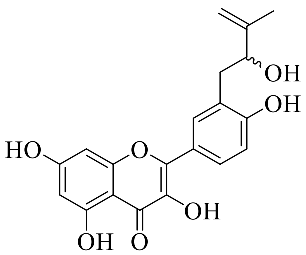

126

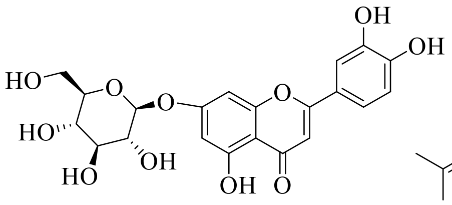

127

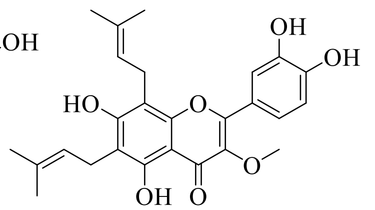

128

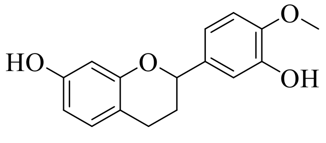

129

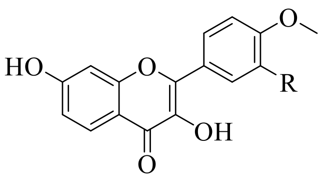

R=H 130
R=OH 131

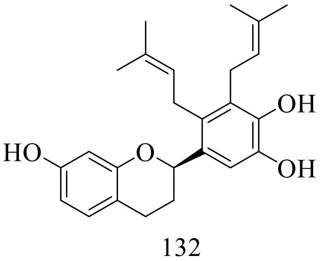

132

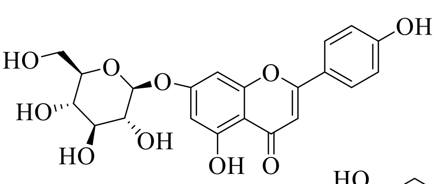

133

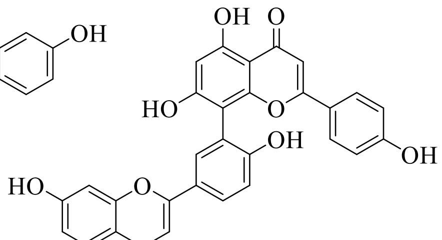

134

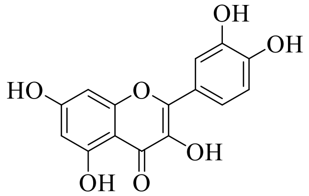

135

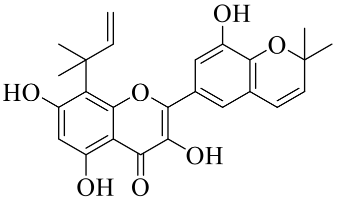

136

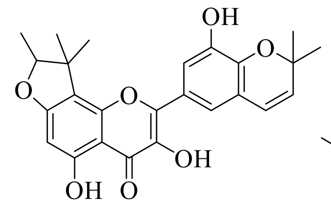

137

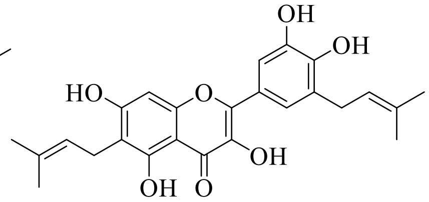

138

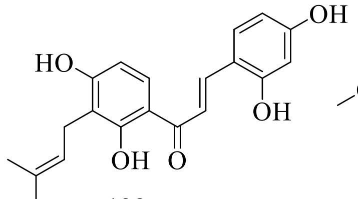

139

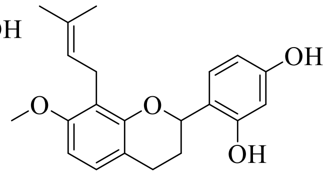

140

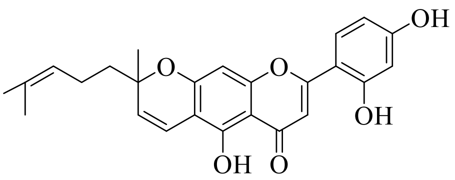

141

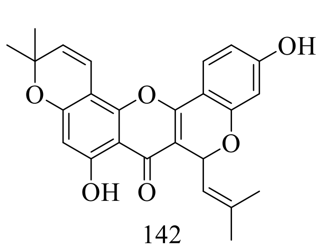

142

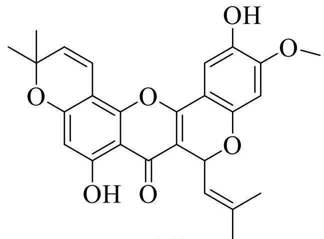

143

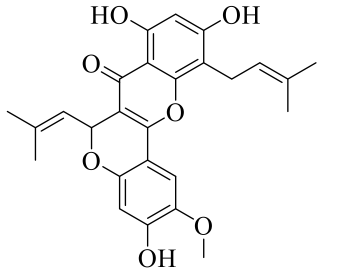

144

Figure S2. Chemical structures of the Penylpropanoids in *Broussonetia* species.

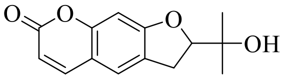

145

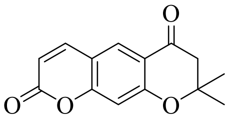

146

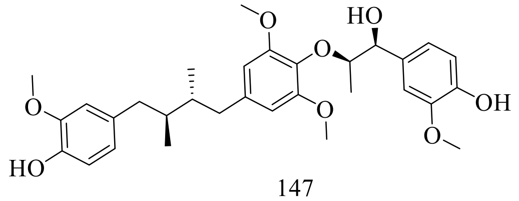

147

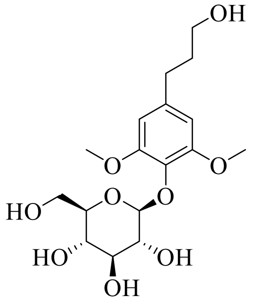

148

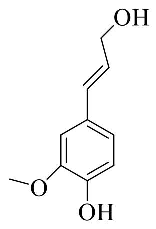

149

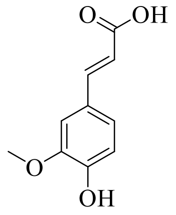

150

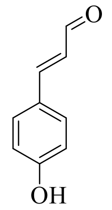

151

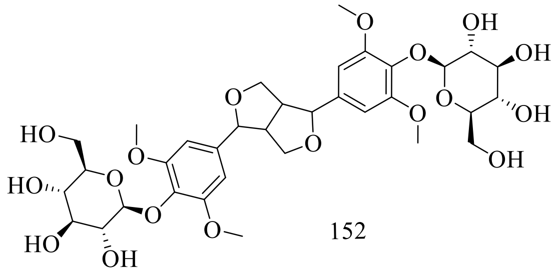

152

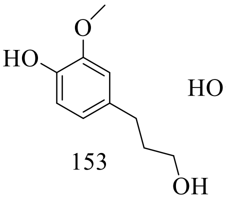

153

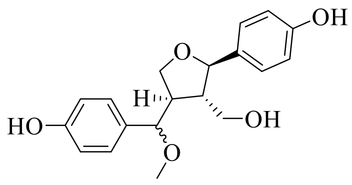

154

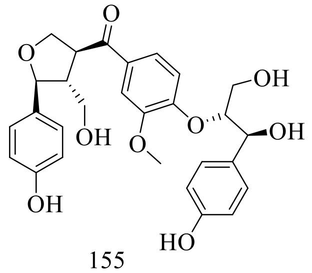

155

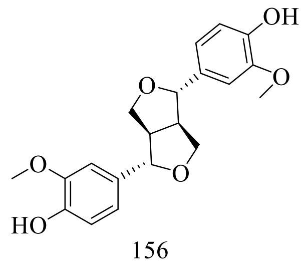

156

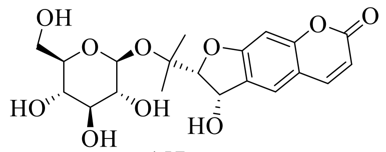

157

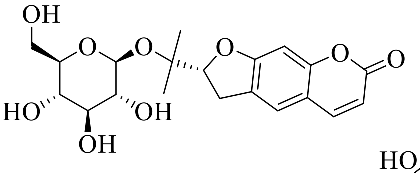

158

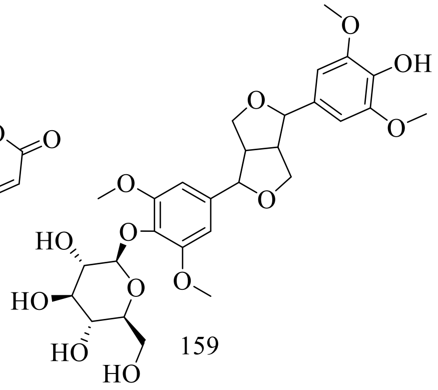

159

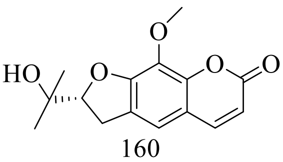

160

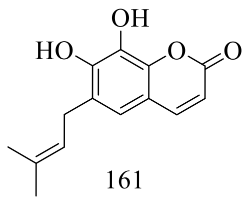
161

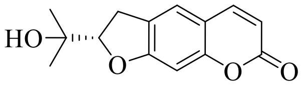
162

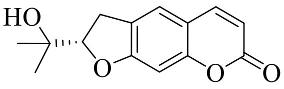
163

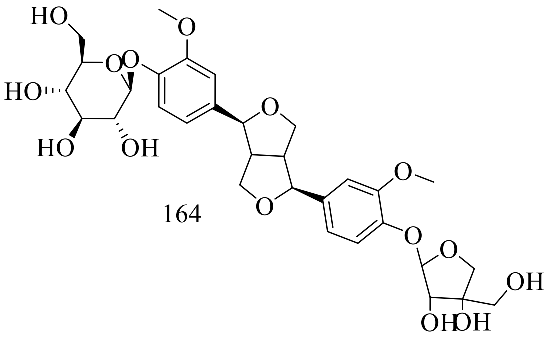
164

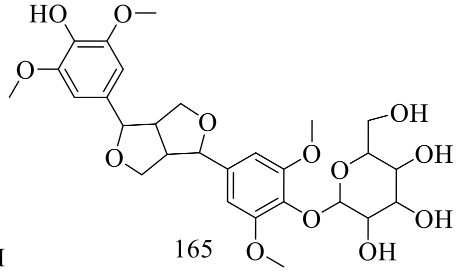
165

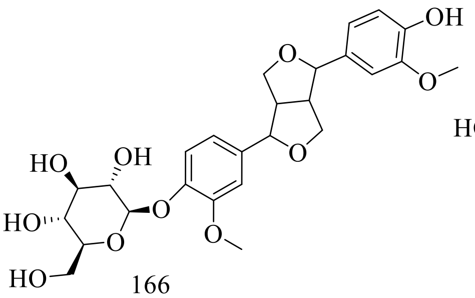
166

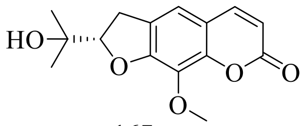
167

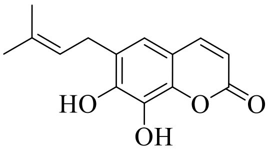
168

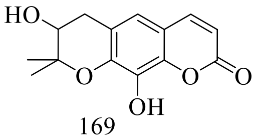
169

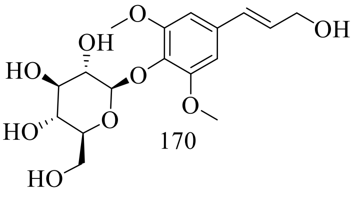
170

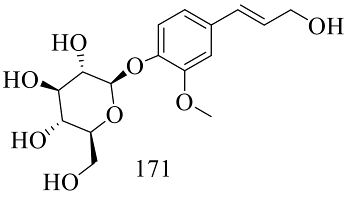
171

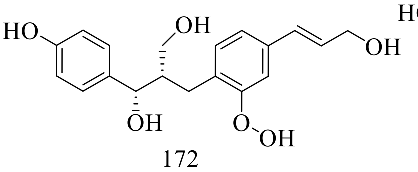
172

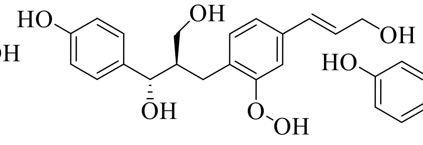
173

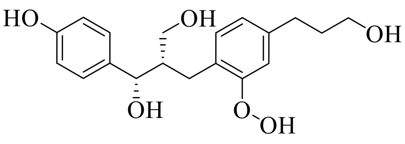
174

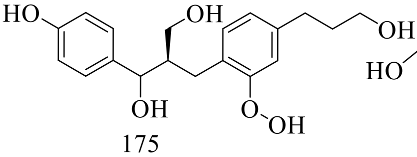
175

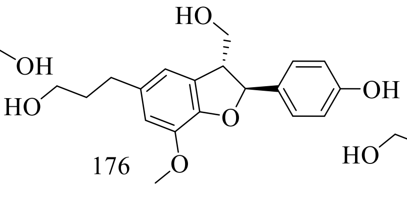
176

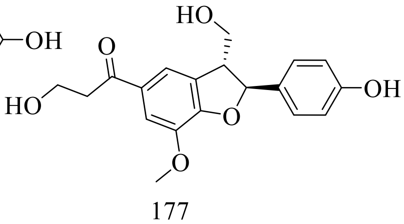
177

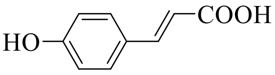

178

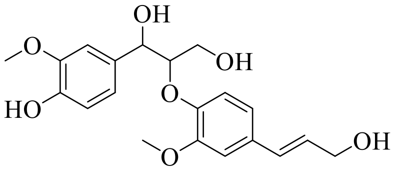

179

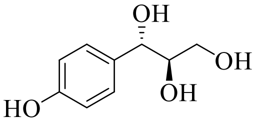

180

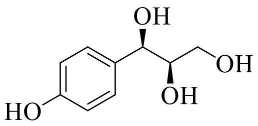

181

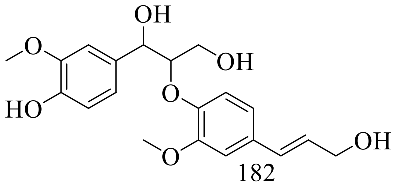

182

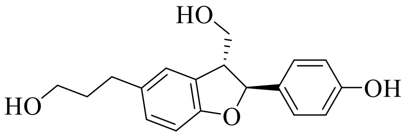

183

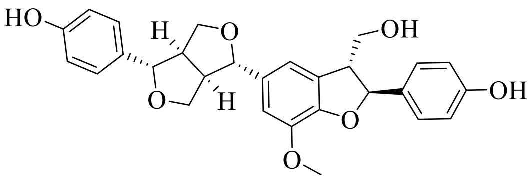

184

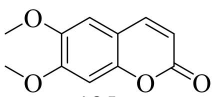

185

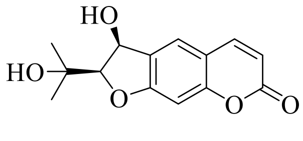

186

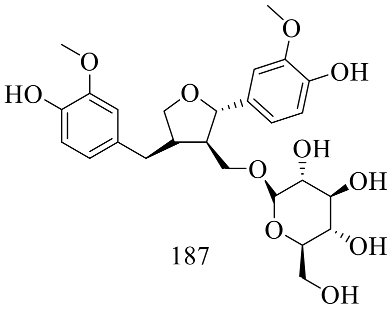

187

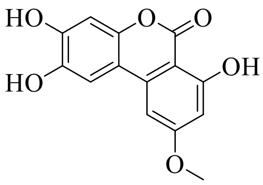

188

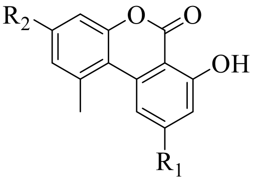

R_1=OH R_2=OCH_3 189
R_1=OCH_3 R_2=OH 190
R_1=OH R_2=OH 191

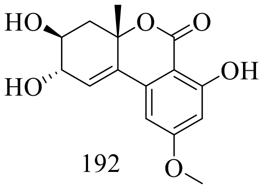

192

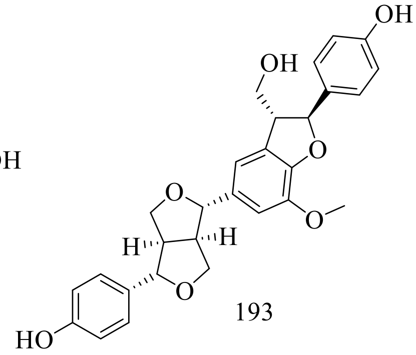

193

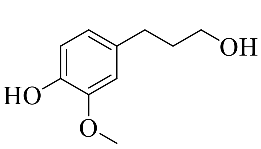

194

Figure S3. Chemical structures of the Polyphenols in *Broussonetia* species.

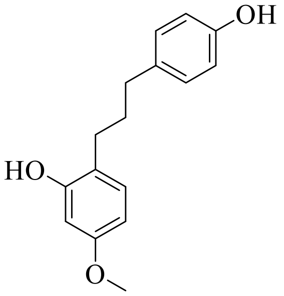

195

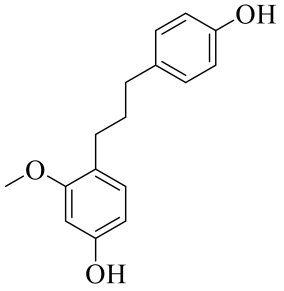

196

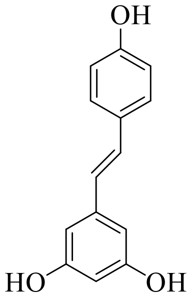

197

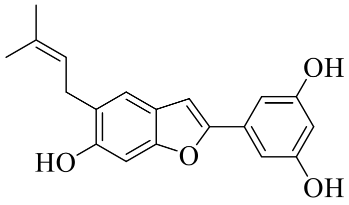

198

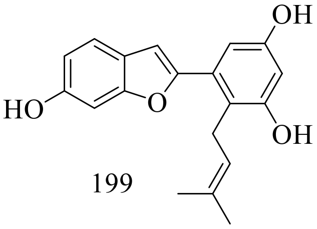

199

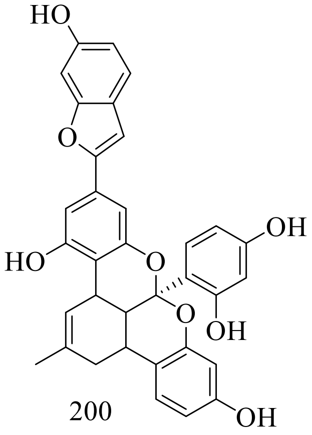

200

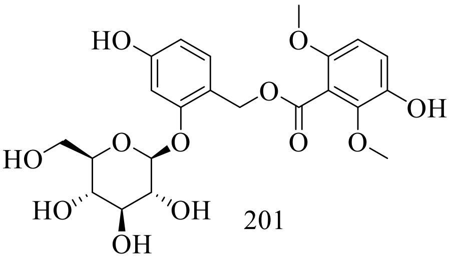

201

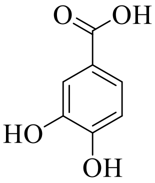

202

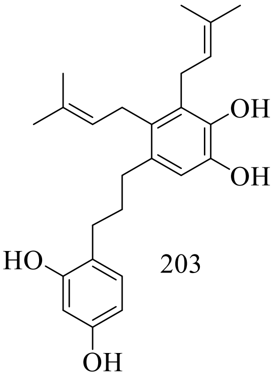

203

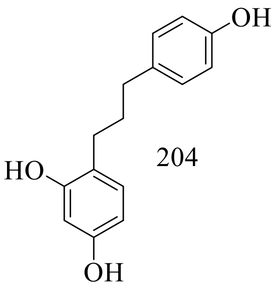

204

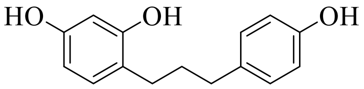

205

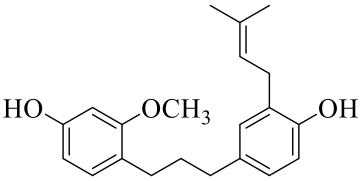

206

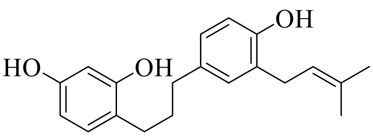

207

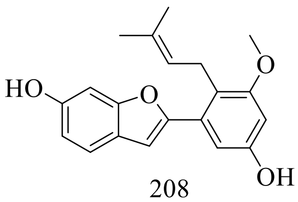

208

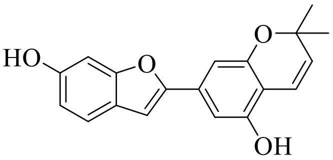

209

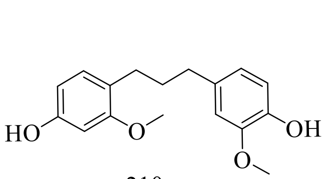

210

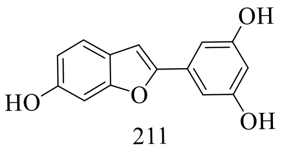

211

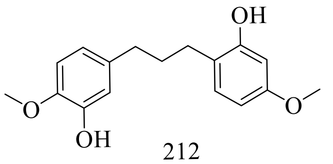

212

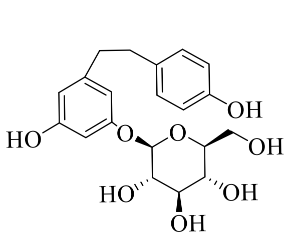

213

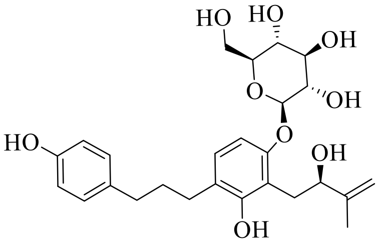

214

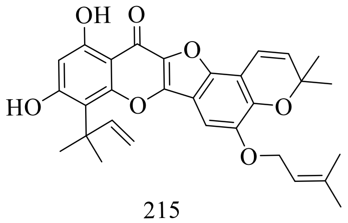

215

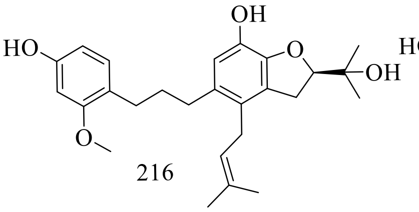

216

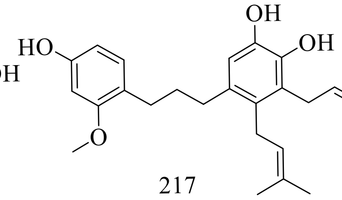

217

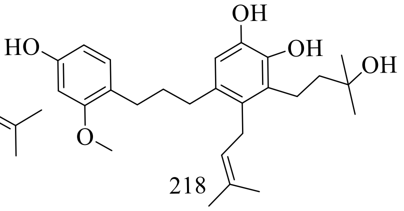

218

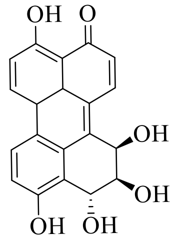

219

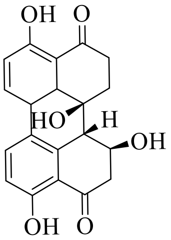

220

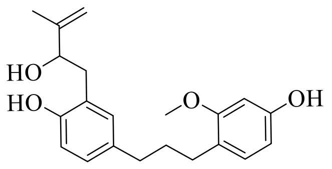

221

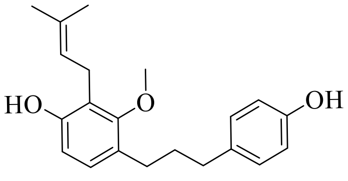

222

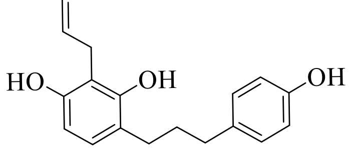

223

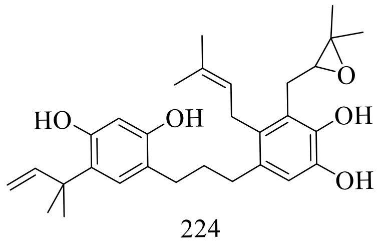

224

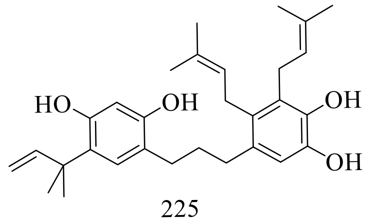

225

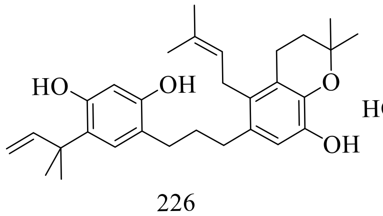

226

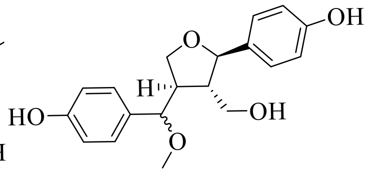

227

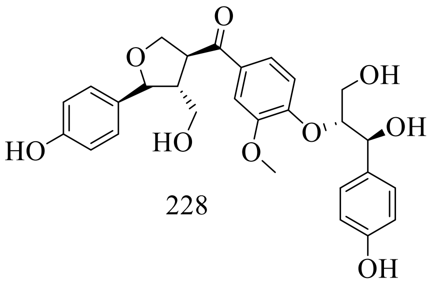

228

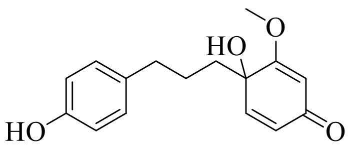

229

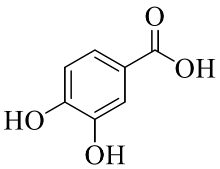

230

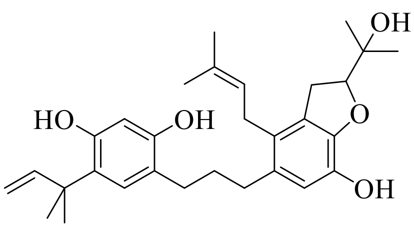

231

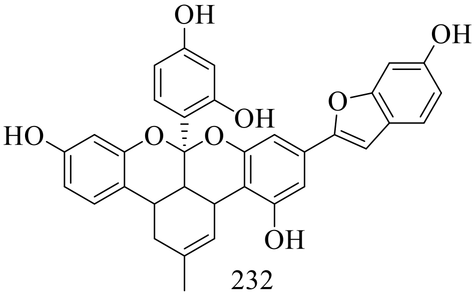

232

Figure S4. Chemical structures of the Alkaloids in *Broussonetia* species.

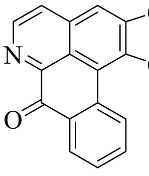
233

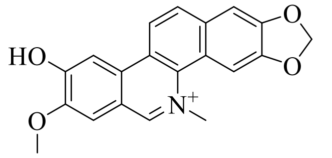
234

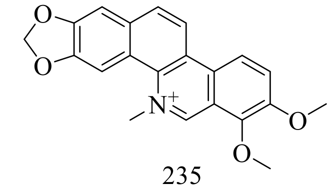
235

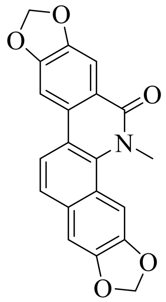
236

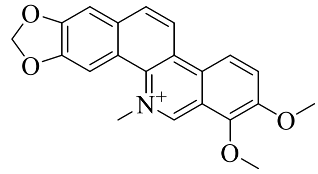
237

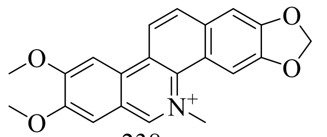
238

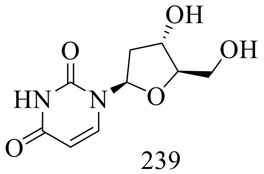
239

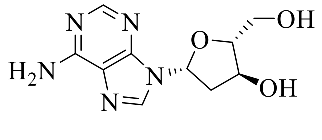
240

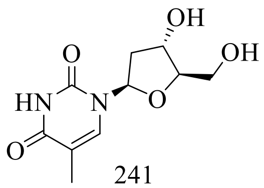
241

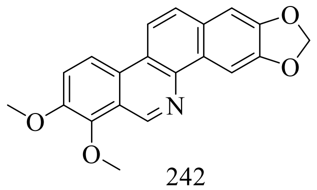
242

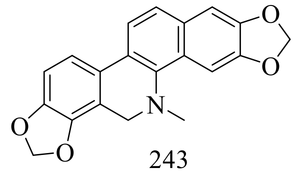
243

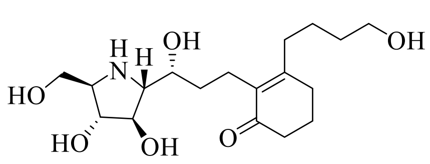
244

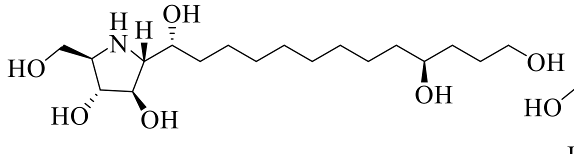
245

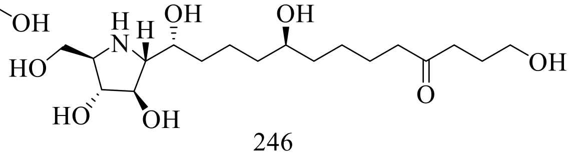
246

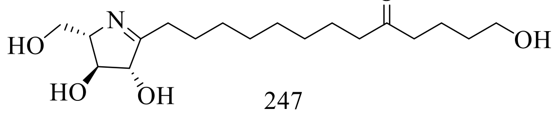
247

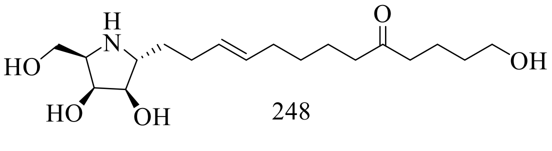
248

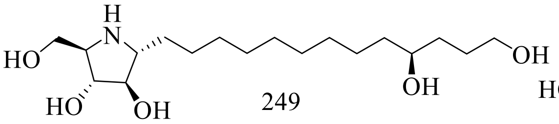

249

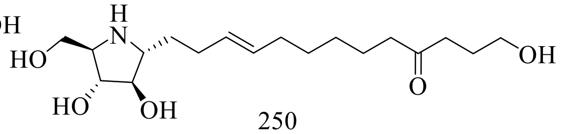

250

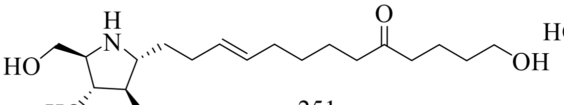

251

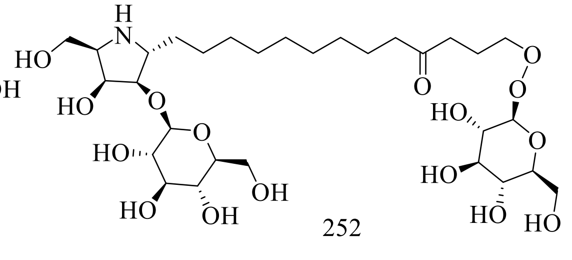

252

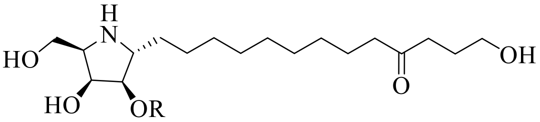

R=β-D-glucopyranosyl 253
R=H 254

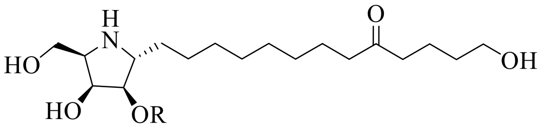

R=β-D-glucopyranosyl 255
R=H 256

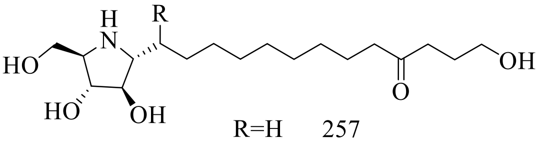

R=H 257
R=OH 258

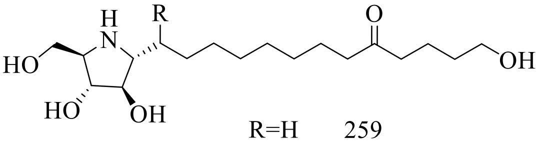

R=H 259
R=OH 260

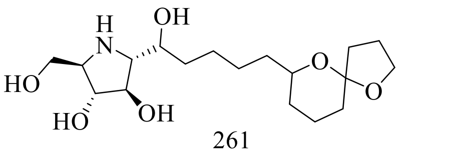

261

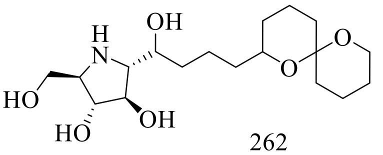

262

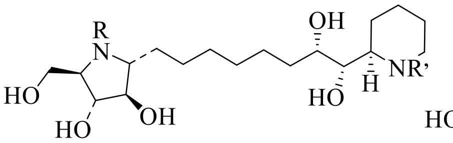

R=H R'=Ac 263
R=R'=Ac 264

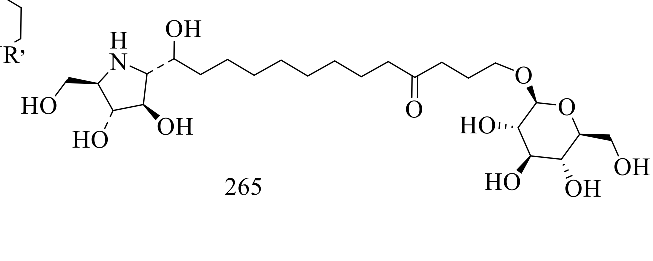

265

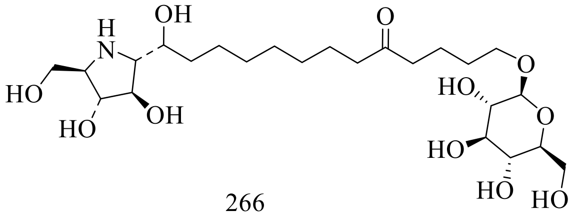

266

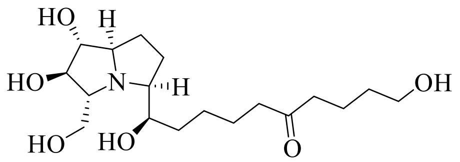

267

Figure S5. Chemical structures of the Terpenoids in *Broussonetia* species.

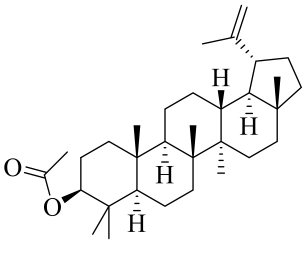

268

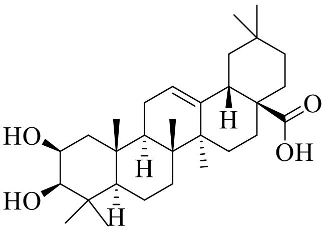

269

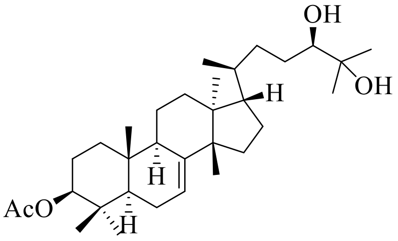

270

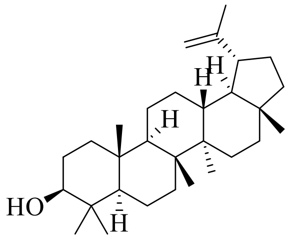

271

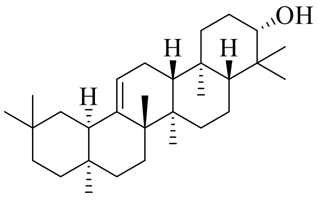

272

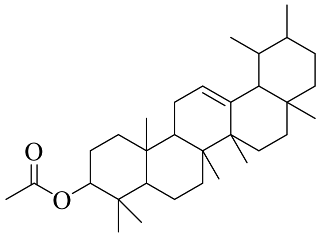

273

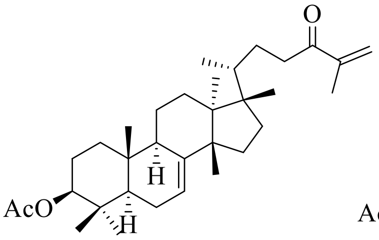

274

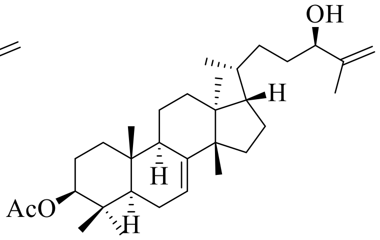

275

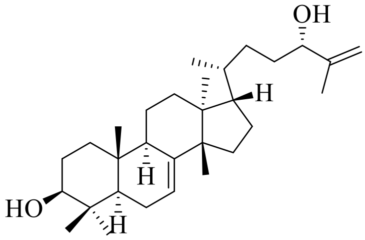

276

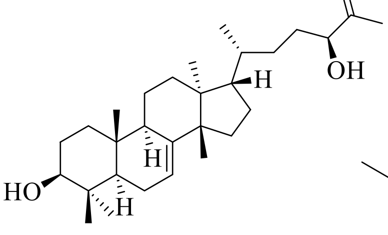

277

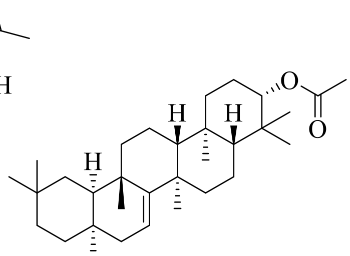

278

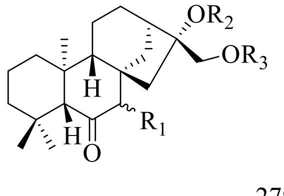

R_1=αOH,R_2=R_3=H 279

R_1=βOH,R_2=R_3=H 280

R_1=αOH,R_2+R_3= 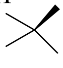 281

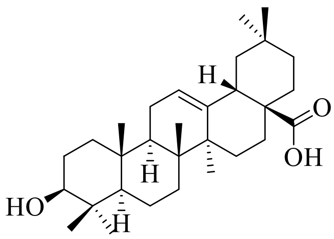

282

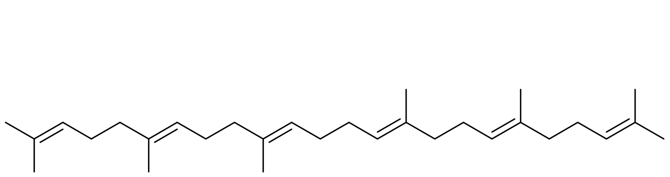

283

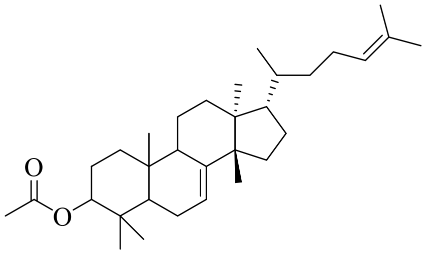

284

Figure S6. Chemical structures of the Steroids in *Broussonetia* species**.**

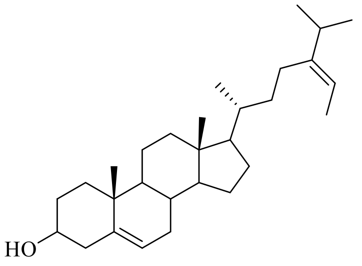

285

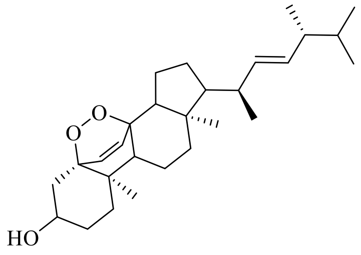

286

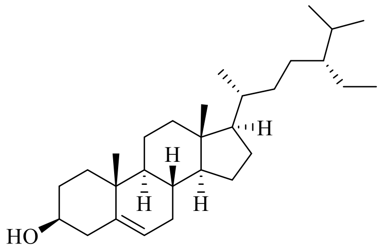

287

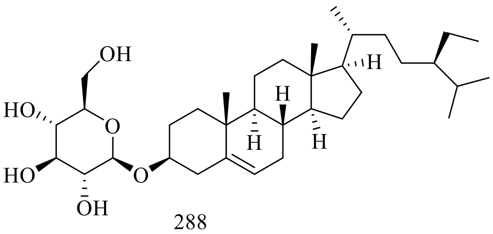

288

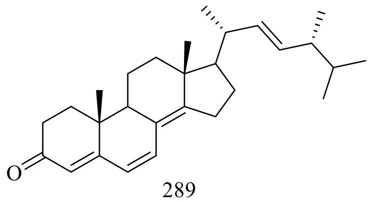

289

Figure S7. Chemical structures of the Others in *Broussonetia* species.

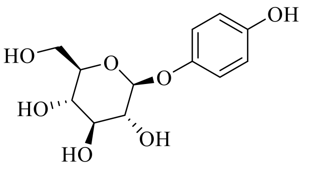

290

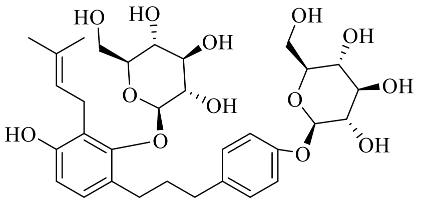

291

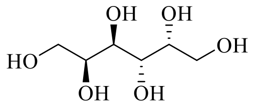

292

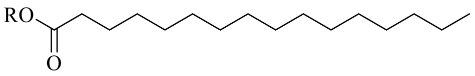

R=Daucosterol 293

R=CH_2CH_3 294

R=H 295

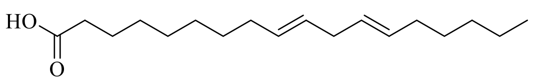

296

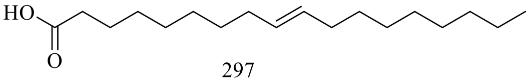

297

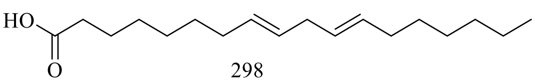

298

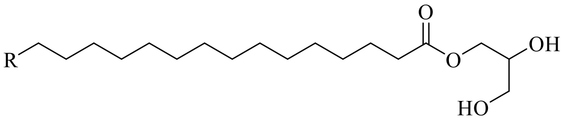

R=CH_3 299

R=CH_2CH_3 300

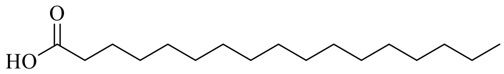

301

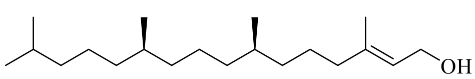

302

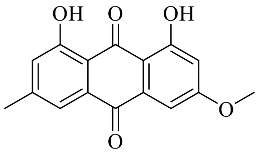

303

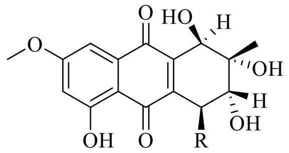

R=OH 304

R=H 305

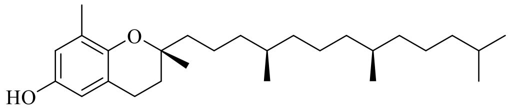

306

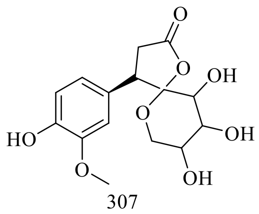

307

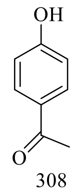

308

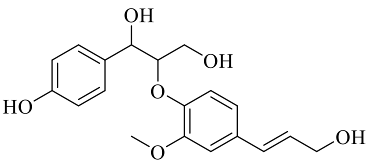

309 310

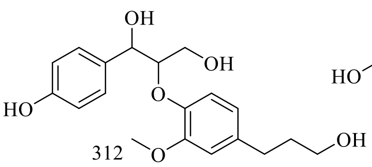

312

311

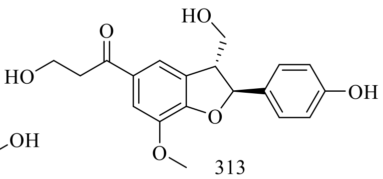

313

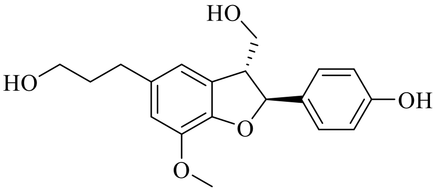
314

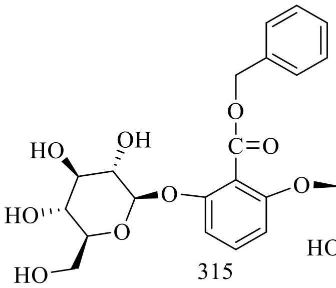
315

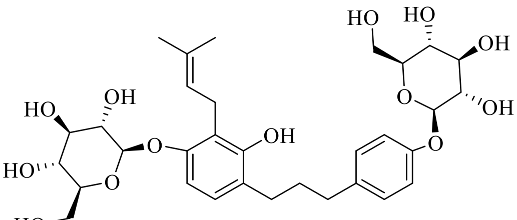
316

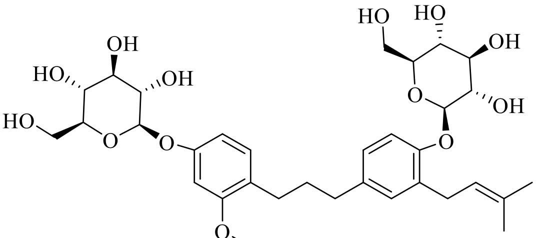
317

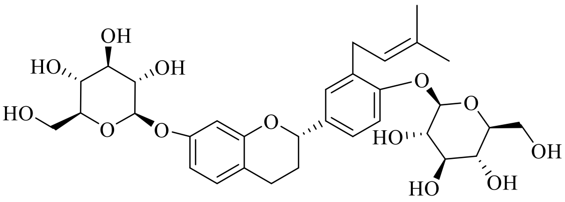
318

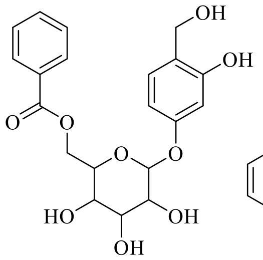
319

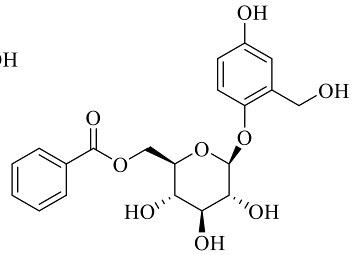
320

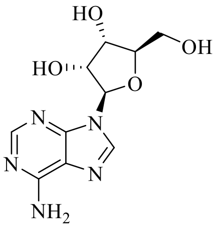
321

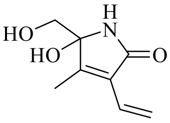
322

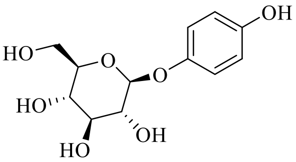
323

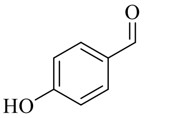
324

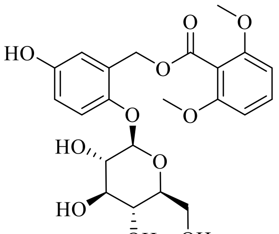
325

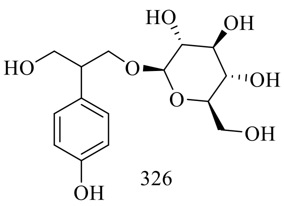
326

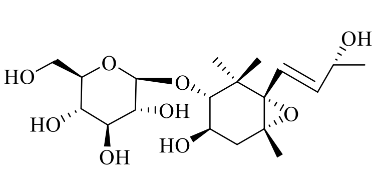
327

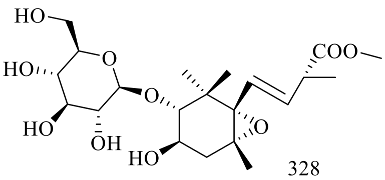

328

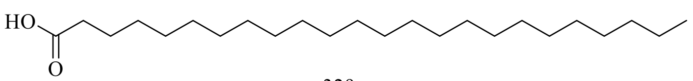

329

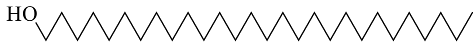

330

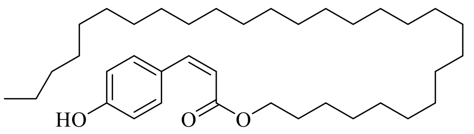

331

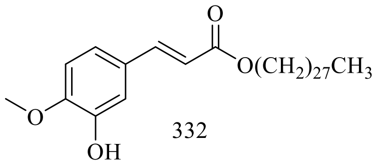

332

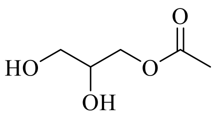

333

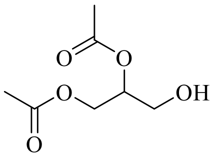

334

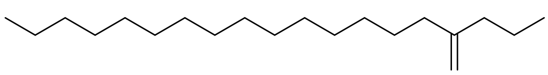

335

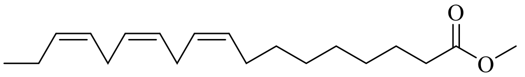

336

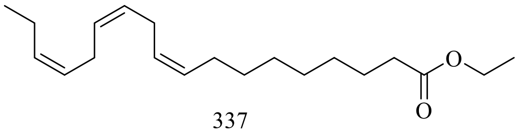

337

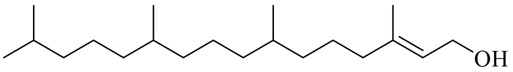

338
